# Prediction of Co and Ru nanocluster morphology on 2D MoS_2_ from interaction energies

**DOI:** 10.3762/bjnano.12.56

**Published:** 2021-07-14

**Authors:** Cara-Lena Nies, Michael Nolan

**Affiliations:** 1 Tyndall National Institute, University College Cork, Lee Maltings, Dyke Parade, Cork, T12 R5CP, Ireland; 2 NIBEC, School of Engineering, University of Ulster at Jordanstown BT37 0QB, United Kingdom

**Keywords:** cobalt (Co), 2D materials, molybdenum disulfide (MoS_2_), ruthenium (Ru), thin film nucleation

## Abstract

Layered materials, such as MoS_2_, have a wide range of potential applications due to the properties of a single layer, which often differ from the bulk material. They are of particular interest as ultrathin diffusion barriers in semiconductor device interconnects and as supports for low-dimensional metal catalysts. Understanding the interaction between metals and the MoS_2_ monolayer is of great importance when selecting systems for specific applications. In previous studies the focus has been largely on the strength of the interaction between a single atom or a nanoparticle of a range of metals, which has created a significant knowledge gap in understanding thin film nucleation on 2D materials. In this paper, we present a density functional theory (DFT) study of the adsorption of small Co and Ru structures, with up to four atoms, on a monolayer of MoS_2_. We explore how the metal–substrate and metal–metal interactions contribute to the stability of metal clusters on MoS_2_, and how these interactions change in the presence of a sulfur vacancy, to develop insight to allow for a prediction of thin film morphology. The strength of interaction between the metals and MoS_2_ is in the order Co *>* Ru. The competition between metal–substrate and metal–metal interaction allows us to conclude that 2D structures should be preferred for Co on MoS_2_, while Ru prefers 3D structures on MoS_2_. However, the presence of a sulfur vacancy decreases the metal–metal interaction, indicating that with controlled surface modification 2D Ru structures could be achieved. Based on this understanding, we propose Co on MoS_2_ as a suitable candidate for advanced interconnects, while Ru on MoS_2_ is more suited to catalysis applications.

## Introduction

Layered materials that can be exfoliated into 2D sheets continue to generate significant interest across various disciplines, including batteries [1–2], catalysis [3–4], electronics [5–10], photonics [11–12], and sensors [13–16]. This is due in part to the interesting properties of these 2D materials, which often differ from their bulk equivalent, as well as the flexibility in fabrication afforded by an ultrathin material [17]. The majority of applications are built on an interaction between a metal and the 2D material. There are multiple studies in this regard that involve the adsorption of or doping with transition metals [4–5,18–22], alkali and alkali earth metals [23–25], and non-metals [25] on MoS_2_ and other 2D materials. While experimental studies can be used to probe the performance of the 2D material in a device or some of the interfacial interactions between metal and 2D materials [9–10,21,26], first principles modelling is a powerful tool that permits the investigation of the detailed interactions of metals and 2D materials at the atomic scale. In particular, understanding the nucleation of metals on 2D materials will be valuable for the design of catalysts or for preventing islanding of conductive metals.

Typically, theoretical studies focus on the adsorption of either single atoms of a series of metals [21,23–25,27] or large nanoparticle-like structures [19–20]. In our previous study we identified that while these studies do deliver useful insights, there is a knowledge gap in the understanding of metal thin film nucleation on 2D materials [28]. We showed that we can investigate the first stages of thin film nucleation on 2D materials with first principles simulations, using the example of small Cu*_n_* structures on an MoS_2_ monolayer (ML).

MoS_2_ is a naturally occurring transition metal dichalcogenide (TMD) and one of the most frequently studied 2D materials. Unlike graphene, MoS_2_ is a semiconductor, which gives it an increased number of possible applications [11,29]. Our previous first principles study [28] of the interaction of Cu species on MoS_2_ showed how Cu can take different structures depending on the number of Cu atoms and whether the TMD is stoichiometric or defective.

In the present study, we will expand the knowledge gained from our previous work on Cu on MoS_2_ and apply it to the adsorption of small Co*_n_* and Ru*_n_* clusters on an MoS_2_ ML, where *n* = 1–4. Co and Ru are of great interest in conjunction with MoS_2_ for application in advanced interconnects as alternatives to Cu [30–35] and TaN. Applications in catalysis include Pt-free hydrogen evolution catalysts [36–41].

Interconnects require high-quality, conformal thin films with low resistivity, to avoid many of the typical failure mechanisms such as electromigration [42–43]. This means that 3D migration of atoms (agglomeration) should be inhibited, while 2D growth (wetting) should be promoted. In contrast, in catalysis applications the ratio of surface to bulk is of great importance in promoting catalytic activity. Therefore, 3D growth (agglomeration) is essential when creating a supported metal catalyst [44–47].

In this work we aim to determine the atomic-scale interactions that control the stability of small Co*_n_* and Ru*_n_* clusters (*n* = 1–4) on a single ML of MoS_2_. Based on this understanding, alongside the magnitude of metal–substrate and metal–metal interactions we will be able to predict the morphology of Co and Ru thin films on 2D MoS_2_. We have previously studied 2D and 3D Cu clusters on TaN, where we determined that there are two useful descriptors for 2D-vs-3D growth [48]: (1) If the metal–substrate interaction is more favourable than the metal–metal interaction, then 2D growth is preferred; and (2) if the total binding energy is more favourable than the cohesive energy of the bulk metal, then 2D growth is preferred.

Predictions made using these descriptors can be used when deciding which metal–substrate combination will be suitable for a particular application where the shape of the metal is vital.

## Methods

All calculations for this study were carried out with density functional theory (DFT) using the Vienna Ab initio Simulation Package (VASP) version 5.4 [49]. Three-dimensional boundary conditions were applied and the spin-polarized general gradient approximation (GGA) along with the Perdew–Burke–Ernzerhof (PBE) approximation to the exchange–correlation functional were used to describe the system [50]. Valence electrons were described explicitly using a plane-wave basis set with an energy cutoff of 450 eV. The valence electron configurations are as follows: Co = 4s^2^ 3d^7^, Ru = 5s^1^ 4d^7^, Mo = 5s^1^ 4d^5^, and S = 3s^2^ 3p^4^. The core electrons were treated with the projector-augmented wave potential (PAW) [51]. A Monkhorst–Pack k-point grid of 2 2 1 was used. All forces acting on the atoms were converged to within 0.02 eV/Å. A Methfessel–Paxton smearing of order 1 was used and no symmetry was applied.

The description of pristine and defective MoS_2_ monolayers (ML) was published in our previous work [28]. Bulk MoS_2_ is made up of two layers. To create the pristine ML one of these was removed, which also creates the vacuum necessary to avoid interaction along the *z*-axis; the vacuum region is 8 Å. A (5 × 5) super cell was used. No van der Waals (vdW) corrections were applied, as both the literature and our own tests (Supporting Information File 1, section S4) show that vdW forces do not dominate in these types of structures. The defective ML has the same structure as the pristine ML, except that a single S atom has been removed to create a vacancy and the ions are relaxed with no symmetry constraints. Using H_2_S as a reference, we have computed an exothermic vacancy formation energy of −6.16 eV. The bond lengths in bulk structures that are used for comparison are based on the crystal structures in [52–57]. Only a theoretical crystal structure was available for RuMo, all other structures used have been determined experimentally.

To understand the binding of Co and Ru to the MoS_2_ monolayer, four different energies are computed:

1. Binding energy per metal atom:

[1] $$E_{\text{bind/atom}}=\frac{\left(E_{\text{total}}-E_{\text{monolayer}}-nE_{\text{metal}\_\text{atom}}\right)}{n}$$

*E*_total_ is the total energy of the relaxed Co*_n_* or Ru*_n_* (*n* = 1–4) adsorbed on MoS_2_. The energy of a single gas-phase metal atom (*E*_metal_atom_) is multiplied by *n*, the number of atoms in adsorbed Co*_n_* or Ru*_n_*.

2. Binding energy with reference to a free Co*_n_* or Ru*_n_* cluster:

[2] $$E_{\text{metal--substrate}}=\frac{E_{\text{total}}-E_{\text{monolayer*}}-E_{\text{metal}\_\text{cluster}}}{n}$$

where *E*_metal_cluster_ is the single point energy of the Co*_n_* or Ru*_n_* nanocluster structure in vacuum. *E*_monolayer*_ is the single point energy of the monolayer after relaxation. We chose to use this instead of the reference used in Equation 1, as surface rearrangements occur in several of the various structures. This method of computing the binding energy isolates the metal–substrate interaction. Using these two methods of computing the binding energy also allows us to determine an approximate metal–metal interaction energy, by applying Equation 3:

3. Metal–metal interaction energy:

[3] $$E_{\text{interact}}=E_{\text{bind/atom}}-E_{\text{metal-substrate}}$$

4. Addition energy:

[4] $$E_{\text{add}}=E_{\text{total}}-E_{\text{monolayer+}(\text{n}-\text{1})\text{metal}}-E_{\text{metal}\_\text{atom}}$$

where *n* is the number of Co or Ru atoms. This models the addition of a metal atom to an existing adsorbed cluster with (*n* − 1) Co or Ru atoms.

## Results and Discussion

### Ru and Co on Pristine MoS_2_

**Figure 1:**
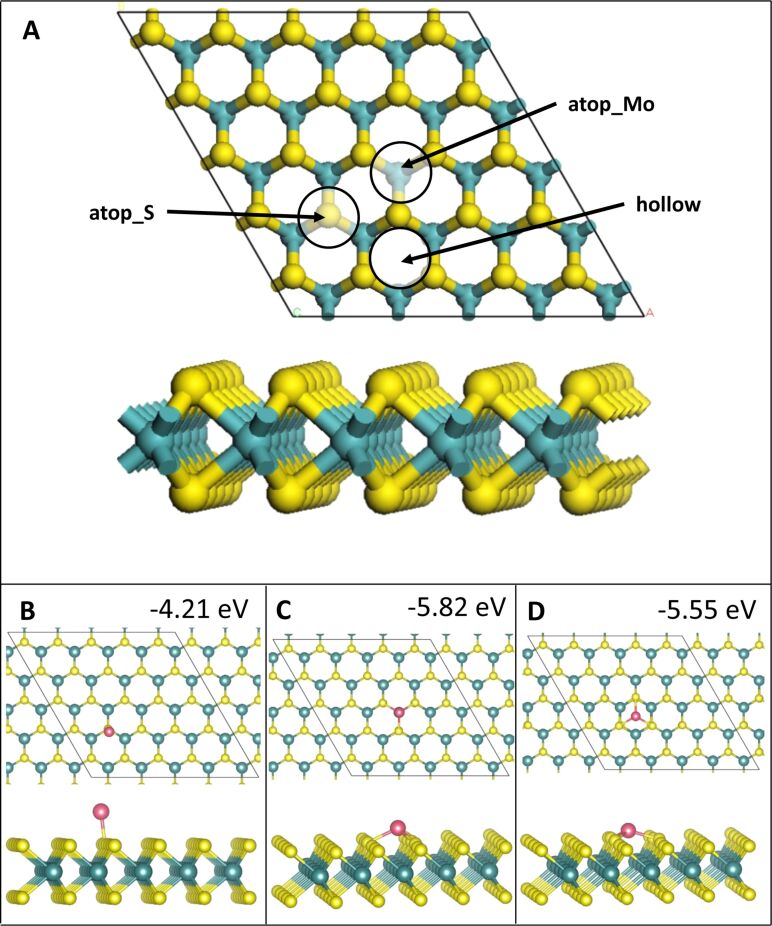
Atomic structure and energies of Co_1_ adsorption modes on perfect MoS_2_. Mo is shown in teal, S in yellow, Co in pink and Ru in purple throughout the article.

As in our previous work [28], there are three metal atom adsorption sites, labelled as **atop_S**, **atop_Mo**, and **hollow**, on the MoS_2_ ML, which are highlighted in Figure 1A. Site **atop_S** has a metal atom adsorbed directly atop a S atom. Site **atop_Mo** has a metal atom binding to three S atoms directly above a Mo atom and site **hollow** has a metal atom binding to three S atoms, but with no Mo atom underneath.

To study how Ru and Co atoms begin to nucleate into a film on the MoS_2_ ML and to compare with the behaviour of Cu on MoS_2_, we adsorb Co*_n_* and Ru*_n_* species with one, two, three, and four atoms on MoS_2_. The binding energies for the set of adsorption structures calculated using Equation 1 are shown in Table 1. Metal–substrate interaction energies calculated from Equation 2 are shown in Table 2, metal–metal interaction energies calculated from Equation 3 are shown in Table 3, and addition energies calculated with Equation 4 are shown in Table S1 of Supporting Information File 1. A structure is considered 2D when all metal atoms are bound directly to the MoS_2_ ML. For 3D structures, at least one of the metal atoms is bound to other metal atoms, but not to MoS_2_.

**Table 1:** Computed binding energies for Co_1_, Co_2_, Co_3_, Co_4_, Ru_1_, Ru_2_, Ru_3_ and Ru_4_ on a MoS_2_ ML for various atom configurations using Equation 1. For the “non-equivalent” configurations for two metal atom adsorption, the column “S_atop site” has atoms at sites S_atop and Mo_atop, “Mo_atop site” has atoms at S_atop and hollow, and “hollow site” has atoms at Mo_atop and hollow.

No. of metal atoms	Configuration	*E*_bind_/Co-atom [eV]			*E*_bind_/Ru-atom [eV]		
		S_atop	Mo_atop	hollow	S_atop	Mo_atop	hollow

1	—	−4.21	−**5.82**	−5.55	−2.47	−3.92	−3.40

2	neighbouring	−6.05	−**6.05**	−5.73	−4.45	−4.45	−3.75
	separated	−4.20	−5.81	−4.92	−2.45	−3.92	−3.19
	non-equivalent	−5.56	−5.37	−5.85	−3.99	−3.71	−3.87

3	line	−5.61	−6.08	−5.88	−4.07	−4.47	−4.41
	triangle	−5.43	−**6.13**	−5.89	−2.57	−4.65	−4.46
	3D triangle	−5.53	−6.05	−5.56	−4.22	−4.72	−4.36

4	line	−6.11	−6.10	−5.95	−4.28	−4.55	−4.76
	rhombus	−5.86	−5.93	−5.87	−4.61	−4.28	−4.37
	3D rectangle	−6.28	−6.33	−5.93	−3.29	−4.93	−4.62
	tetrahedral	−5.85	−6.32	−6.05	−4.70	−5.14	−4.74

**Table 2:** Computed metal–substrate interaction energies for Co_2_, Co_3_, Co_4_, Ru_2_, Ru_3_ and Ru_4_ on a MoS_2_ ML for various atom configurations using Equation 2. For the “non-equivalent” configurations for two metal atom adsorption, the column “S_atop site” has atoms at sites S_atop and Mo_atop, “Mo_atop site” has atoms at S_atop and hollow, and “hollow site” has atoms at Mo_atop and hollow.

No. of metal atoms	Configuration	*E*_metal−substrate_/Co-atom [eV]			*E*_metal−substrate_/Ru-atom [eV]		
		S_atop	Mo_atop	hollow	S_atop	Mo_atop	hollow

2	neighbouring	−2.71	−2.64	−2.97	−2.71	−2.67	−4.55
	separated	−1.47	−3.00	−2.05	0.58	−1.21	−0.60
	non-equivalent	−1.44	−1.54	−2.02	−1.61	−1.36	−3.96
							
3	line	−1.17	−2.78	−4.78	−0.79	−3.17	−5.14
	triangle	−1.66	−2.31	−2.29	0.12	−2.07	−2.61
	3D triangle	−0.77	−1.42	−0.92	−0.66	−1.32	−1.88
							
4	line	−1.79	−2.46	−3.77	−0.16	−14.10	−4.65
	rhombus	−0.69	−0.98	−3.42	0.62	1.01	−0.86
	3D rectangle	−1.69	−1.55	−1.50	−0.24	−1.24	−1.49
	tetrahedral	−0.85	−1.69	−2.40	−0.79	−1.67	−5.19

**Table 3:** Computed metal–metal interaction energies using Equation 3. For the “non-equivalent” configurations for two metal atom adsorption, the column “S_atop site” has atoms at sites S_atop and Mo_atop, “Mo_atop site” has atoms at S_atop and hollow, and “hollow site” has atoms at Mo_atop and hollow.

No. of metal atoms	Configuration	*E*_interact_/Co-atom [eV]			*E*_interact_/Ru-atom [eV]		
		S_atop	Mo_atop	hollow	S_atop	Mo_atop	hollow

2	neighbouring	−3.34	−3.42	−2.76	−1.74	−1.77	0.79
	separated	−2.73	−2.82	−2.88	−3.04	−2.71	−2.59
	non-equivalent	−4.11	−3.84	−3.84	−2.38	−2.34	−0.09

3	line	−4.43	−3.30	−1.09	−3.28	−1.30	0.73
	triangle	−0.98	−3.82	−3.60	−2.68	−2.58	−1.86
	3D triangle	−4.77	−4.63	−4.64	−3.56	−3.40	−2.48
							
4	line	−4.32	−3.64	−2.18	−4.12	9.56	−0.11
	rhombus	−5.17	−4.95	−2.45	−5.23	−5.29	−3.50
	3D rectangle	−4.59	−4.79	−4.43	−3.05	−3.69	−3.13
	tetrahedral	−5.00	−4.63	−3.66	−3.92	−3.48	−4.72

**Figure 2:**
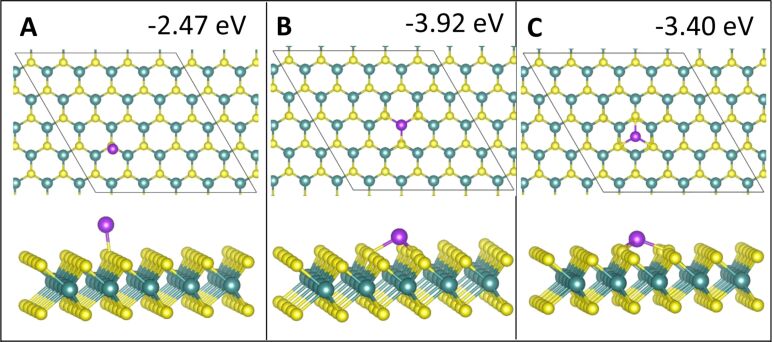
Atomic structure and energies of Ru_1_ adsorption modes on perfect MoS_2_.

**Figure 3:**
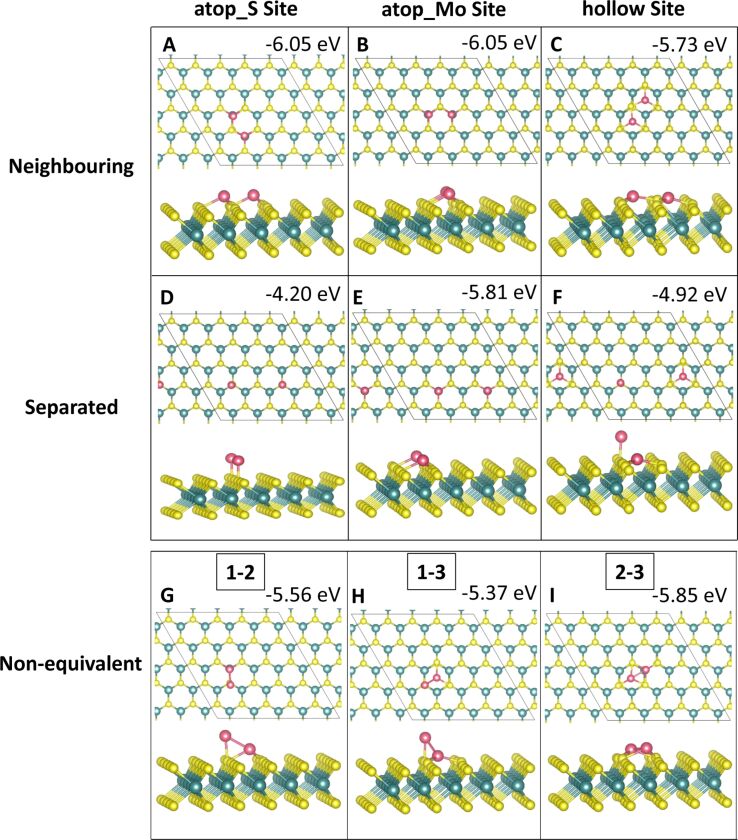
Atomic structure and energies of Co_2_ adsorption modes on perfect MoS_2_.

**Figure 4:**
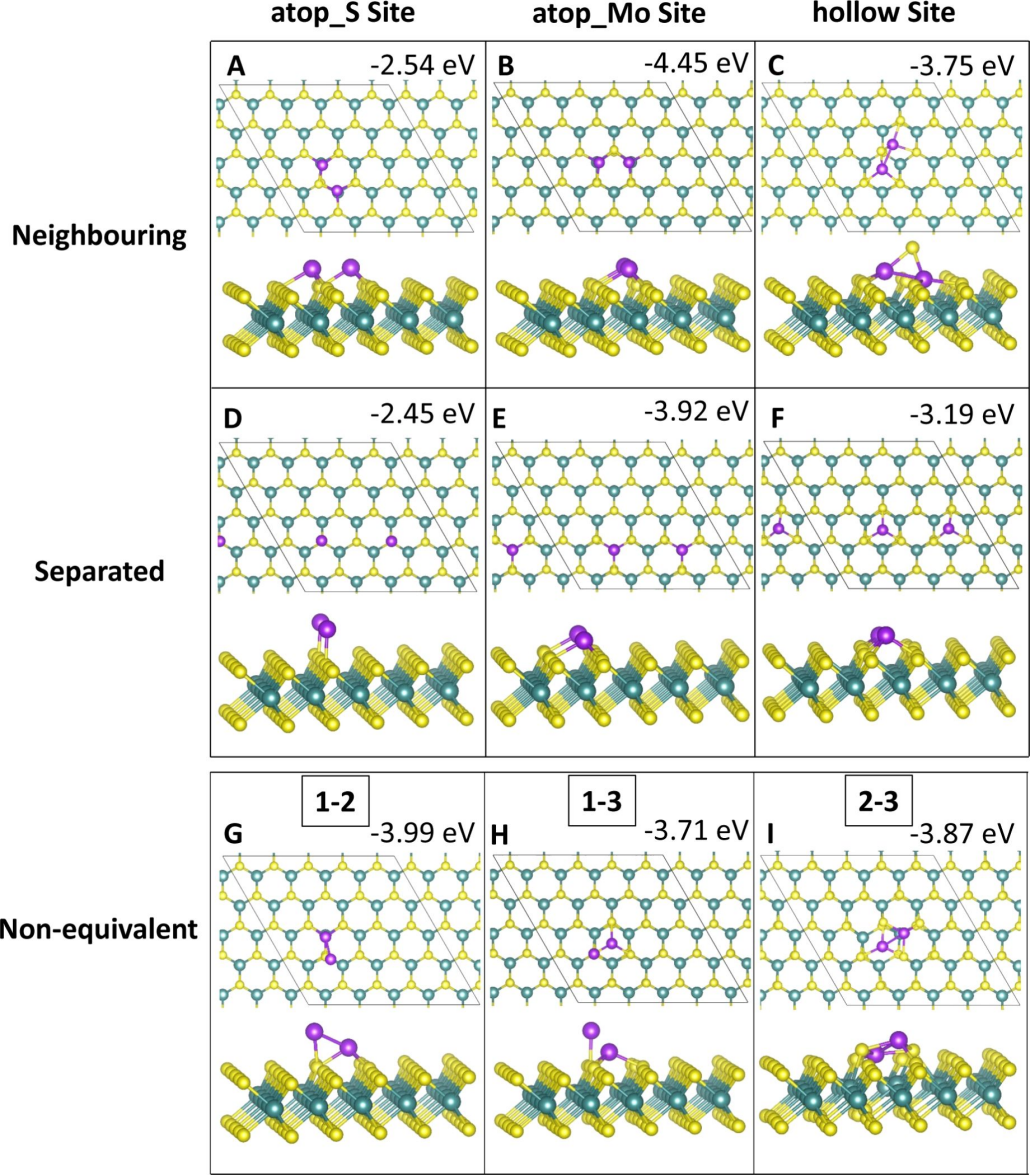
Atomic structure and energies of Ru_2_ adsorption modes on perfect MoS_2_.

**Figure 5:**
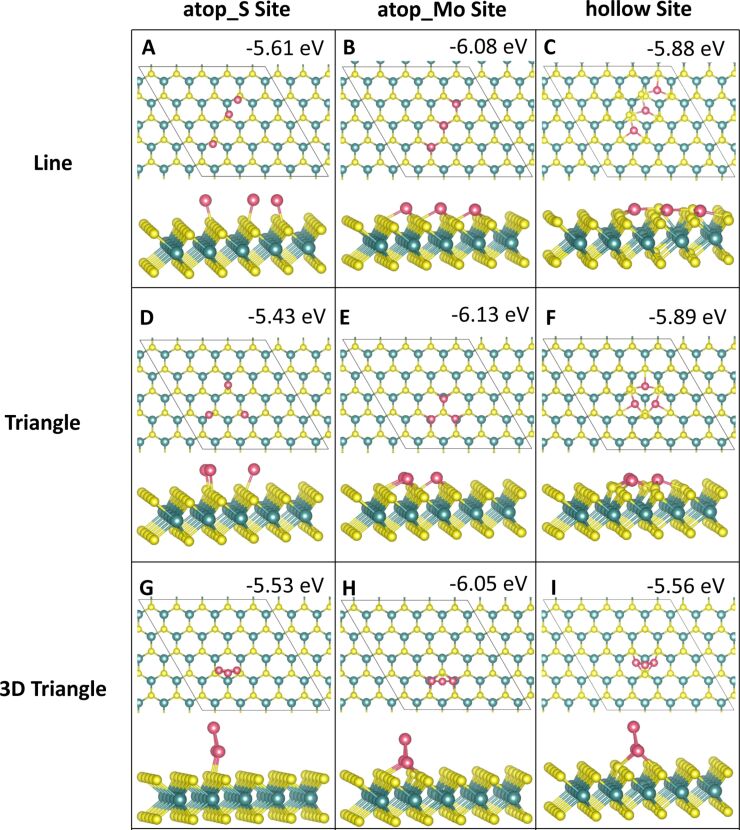
Atomic structure and energies of Co_3_ adsorption modes on perfect MoS_2_.

**Figure 6:**
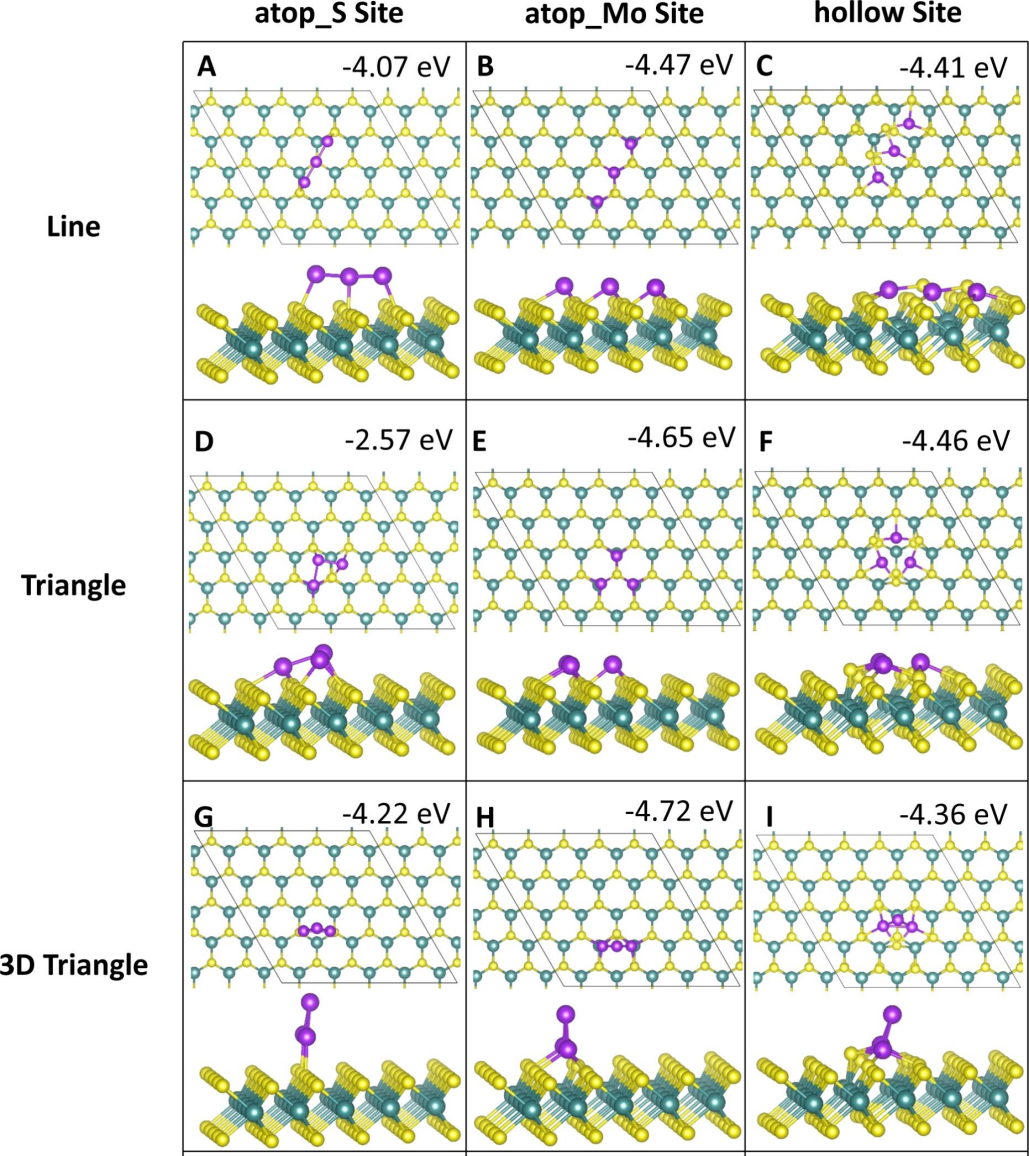
Atomic structure and energies of Ru_3_ adsorption modes on perfect MoS_2_.

**Figure 7:**
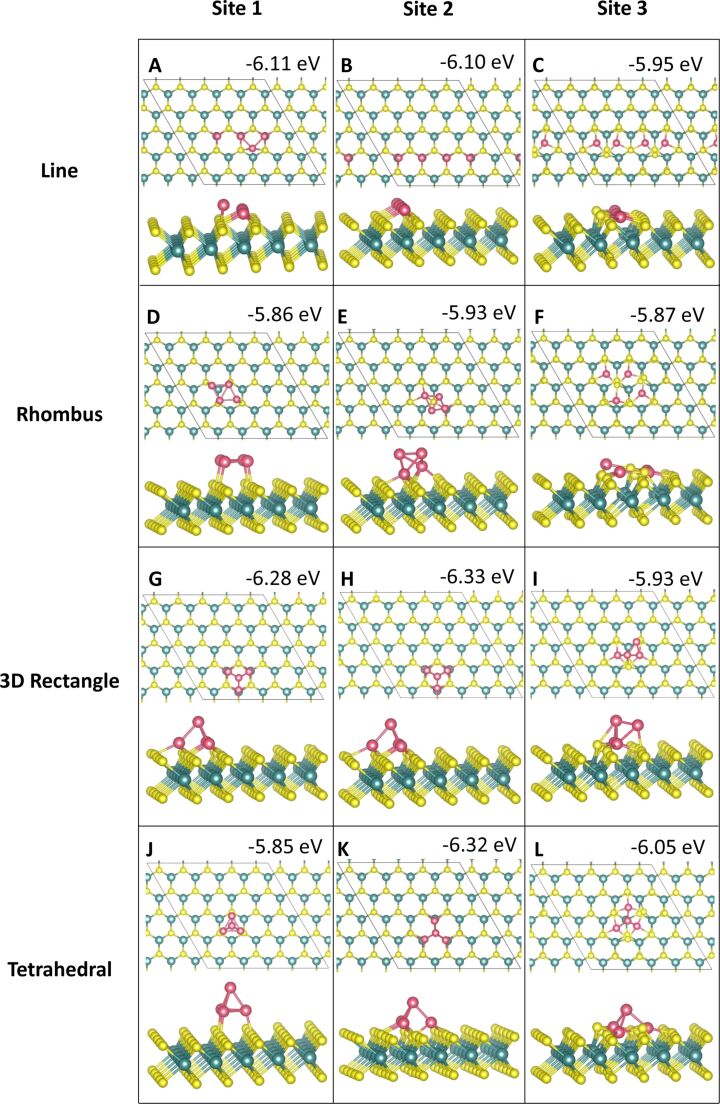
Atomic structure and energies of Co_4_ adsorption modes on perfect MoS_2_.

**Figure 8:**
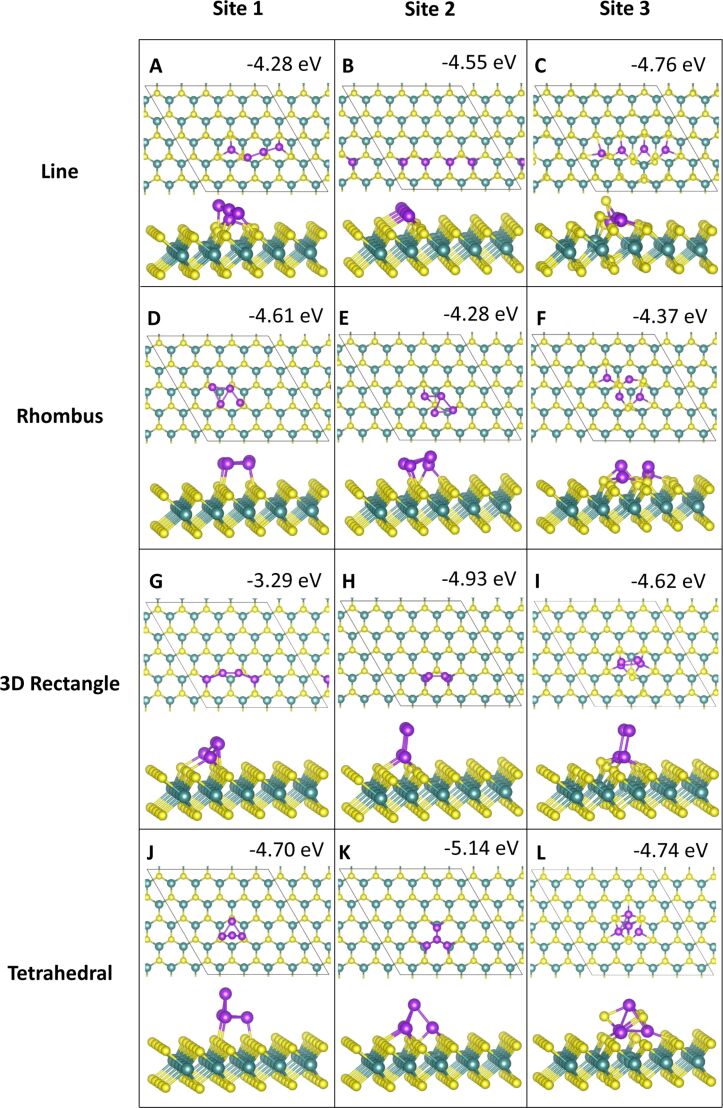
Atomic structure and energies of Ru_4_ adsorption modes on perfect MoS_2_.

The relaxed geometries of the various Co*_n_* species are shown in Figure 1 and below in Figure 3, Figure 5, and Figure 7, while the geometries for Ru*_n_* species are shown below in Figure 2, Figure 4, Figure 6, and Figure 8. The energies shown in these figures are the binding energies computed using Equation 1. Structures are referred to and labelled according to the initial cluster adsorption structure, to avoid confusion due to any geometry rearrangements that occur.

In the following sections we elaborate how factors influencing structure stability vary as the cluster size increases. Such factors include the presence or absence of metal–metal bonds, symmetrical versus asymmetrical addition, incorporation of atoms into the ML, adsorption sites, and changes to stability in the presence of an S vacancy. Our findings are supported through analysis of geometry variations, bond lengths, adsorption, addition and metal–metal interaction energies, and Bader charges.

#### Single-atom adsorption

Co and Ru atoms adsorb exothermically at all three adsorption sites. A single Co atom adsorbs most strongly at site **atop_Mo**, with an energy gain of −5.82 eV, followed by site **hollow** and site **atop_S**, with energy gains of −5.55 eV and −4.21 eV, respectively. A single Ru atom adsorbs preferentially at site **atop_Mo** with an energy gain of −3.92 eV, followed by site **hollow** and site **atop_S**, with energy gains of −3.40 eV and −2.47 eV, respectively.

Similarly to Cu, for both Co and Ru, site **atop_Mo** is the most favourable adsorption site. This is likely due to the adatom position repeating the geometry of the MoS_2_ ML. We find that for a single adatom, Co–S distances are the shortest of all the metals studied, lying between 1.99 and 2.16 Å depending on the adsorption site. These Co–S distances are shorter than the Co–S distance of 2.31 Å in bulk CoS_2_ [53]. Similarly, the Ru–S distances are between 2.13 and 2.24 Å, compared to 2.37 Å in bulk RuS_2_ [56]. The shortest metal–S bonds are measured at site **atop_S**, compared to those measured at sites **atop_Mo** and **hollow**. There are some Co–Mo bonds observed, with Co–Mo distances of 2.85 Å, but there are no Ru–Mo bonds or Cu–Mo bonds [28]; the presence of these could be one origin for the enhanced interaction between Co and the MoS_2_ ML. The relaxed geometries for adsorption of Co_1_ and Ru_1_ are shown in Figure 1B–D and Figure 2, respectively.

#### Two-atom adsorption

For two-adatom adsorption, there are three different M_2_ geometries. All of these are 2D. They involve atoms adsorbed at nearest neighbour equivalent surface sites, at equivalent but separated sites and atoms at neighbouring but non-equivalent sites. Relaxed geometries for adsorption of Co_2_ and Ru_2_ are shown in Figure 3 and Figure 4, respectively.

For Co, adsorbing two atoms at equivalent neighbour sites corresponding to adsorption site **atop_Mo** is the most favourable Co_2_ configuration, with a computed adsorption energy of −6.05 eV/atom. Adatoms originally adsorbed as equivalent neighbours at site **atop_S** migrate to site **atop_Mo** instead (Figure 3A,B). This adsorption mode is more favourable by 0.32 eV compared to equivalent neighbours at site **hollow** (Figure 3C).

The adsorption of two Co atoms is preferred at equivalent, separated sites corresponding to adsorption mode **atop_Mo** (Figure 3E). We propose that this is due to the geometry match with the substrate as there are no Co–Co bonds to contribute to the binding energy, only the weaker adsorption has Co–Mo bonds and both adsorption sites have metal interaction energies within less than 0.1 eV of each other. Adsorption of separated atoms at site **atop_S** is the least favourable configuration for Co_2_ (Figure 3D).

Starting from a configuration of Co_2_ adsorbed at site **hollow**, during relaxation one Co atom migrated to site **atop_S**, making this a non-equivalent adsorption at separated sites **atop_S - hollow**, as shown in Figure 3F. This explains the less favourable binding energy of −4.92 eV compared to −5.73 eV at equivalent neighbouring sites **hollow** and −5.37 eV at non-equivalent neighbouring sites **atop_S - hollow**.

Adsorption of Co atoms at neighbouring but non-equivalent sites is overall more favourable than adsorption of separated atoms, in particular where the Co atoms bind to sites **atop_Mo - hollow** (Figure 3I). These structures also have the strongest metal–metal interaction energies (Table 3). This is due to Co–Co bonding, which is present exclusively for these Co_2_ adsorption modes due to the decreased distance between atoms compared to equivalent sites. We also observe large addition energies for these structures, showing that addition of a second atom can stabilise any atoms that may be initially adsorbed at a less favourable site.

During the adsorption of Ru_2_, several rearrangements were observed. While adsorption of two neighbouring equivalent Ru atoms at site **atop_Mo** is most favourable (Figure 4B), with *E*_bind_ = −4.45 eV, the initial adsorption at neighbouring equivalent sites **atop_S** resulted in both atoms migrating to site **atop_Mo** (Figure 4B), similar to Co_2_, indicating that this particular configuration is unstable for metal atoms adsorbed at site **atop_S**. The adsorption of Ru at separated sites **atop_S** (Figure 4D) is the least favourable configuration for Ru_2_, as well, although no rearrangements or migration occur here.

Similar to Co_2_, adsorption of Ru at equivalent separated sites **atop_Mo** (Figure 4E) is the second most favourable of the equivalent Ru_2_ adsorptions. The large distortions of the MoS_2_ ML for an initial configuration of neighbouring Ru at site **hollow** (Figure 4C), are caused by the attempted incorporation of one Ru atom into the S layer during relaxation. This has also caused an S atom adjacent to Ru to move out of the surface and bond to both Ru atoms from above, forming a triangular Ru_2_S structure. With a binding energy of −3.99 eV, the combination of sites **atop_S - atop_Mo** (Figure 4G) is as favourable as separated atoms at site **atop_Mo** (Figure 4E). This indicates, that at this very early stage of film growth there is no preference yet between structures with separated atoms and those with Ru–Ru bonds.

Those configurations that underwent strong rearrangement during relaxation, that is, neighbouring equivalent atoms at site **hollow** (Figure 4C) and neighbouring atoms at the non-equivalent site **atop_Mo - hollow** (Figure 4I) have very weak metal–metal interaction energies (Table 3). The neighbouring site **hollow** has a positive metal–metal interaction energy, while the non-equivalent site **atop_Mo - hollow** has an interaction energy close to zero. The latter should mean that metal atoms do not agglomerate or separate, while a positive metal–metal interaction should be indicative of a separation of atoms. However, a zero or positive metal–metal energy can only be computed when *E*_bind*_, calculated from Equation 2, is larger than or almost equal to *E*_bind_, calculated from Equation 1. As *E*_bind*_ is calculated using the structure of the MoS_2_ ML after metal relaxation and the energy of the Ru cluster in vacuum as references, this indicates that for these structures *E*_bind_ reflects energy changes during rearrangement, which in turn affects the magnitude of the metal–metal interaction energy.

#### Three-atom adsorption

Three-atom adsorption involved the study of three different geometries, two of which are 2D and one of which is 3D. These are shown in Figure 5 and Figure 6. For the first configuration, the three adatoms are adsorbed in a line along neighbouring, equivalent sites. In the other two configurations the adatoms are arranged in a triangle, one 2D on the surface and the other in a 3D triangle; in the latter, the third adatom sits atop the two other adatoms.

The most favourable configuration for Co_3_ is the 2D triangle at site **atop_Mo** with a computed adsorption energy of −6.13 eV/atom (Figure 5E). The line and 3D triangle configurations are, however, competitive in energy. The line configuration is more favourable by between 0.01 and 0.32 eV depending on the initial site at which the Co atoms bind. The 2D triangle with Co atoms at site **atop_S** has the least favourable adsorption energy of −4.22 eV/atom (Figure 5D). This configuration also has the weakest metal–metal interaction energy of −0.98 eV, as the Co atoms move away from each other. The Co–Co distances are 2.08 Å between the adjacent Co atoms and 4.89 to 4.98 Å to the separated atom.

The largest metal–metal interaction energies, all of which are greater than 4 eV in magnitude, can be observed for all 3D triangle configurations (Figure 5G–I) as well as for the line configuration at site **atop_S** (Figure 5A). This is due to the presence of Co–Co bonds in all of these configurations, as there is no metal–metal bonding observed in the other structures. The Co–Co distances are between 2.20 and 2.25 Å, with some few longer bonds of approx. 2.6 Å compared to 2.48 Å in bulk Co[52].

For Ru_3_, a different trend is seen. Here, the most favourable adsorption occurs for the 3D triangle configuration with the Ru atoms at site **atop_Mo** (Figure 6H). However, all Ru_3_ configurations, with the exception of the 2D triangle configuration at site **atop_S**, with an adsorption energy of −2.57 eV/atom (Figure 6D), differ by no more than 0.7 eV/atom from this. This least favourable adsorption structure displays Ru atoms that migrate significantly from their original adsorption sites. While one atom remains at site **atop_S**, the second atom bridges between two S atoms between site **atop_Mo** and site **hollow**. The third atom has migrated to bind at site **atop_Mo**. Even though there are Ru–Ru bonds, this structure is distorted, compared to the more stable structures, leading to a less favourable adsorption energy.

Compared to Co_3_, the metal–metal interactions are not as strong for Ru_3_. Similarly to Co_3_, the strongest metal–metal interactions are found for those structures where Ru–Ru bonding is present. Similar to Ru_2_, adsorption in a linear configuration, at site **hollow** (Figure 6C), yields a positive interaction energy. The reason for this are the significant distortions to the ML, in which S atoms migrate out of the surface. Similar rearrangements can be observed for all Ru_3_ structures at site **hollow** (Figure 6C,F,I) as well as for the 2D Co_3_ structures at site **hollow** (Figure 5C,F). Compared to copper, where we did not observe this distortion, the smaller size of Co and Ru compared to Cu may promote these distortions when the metal atoms are adsorbed above the hollow site **hollow**. In response the S atoms can migrate and rearrange to accommodate the additional metal atom.

#### Four-atom adsorption

For four-atom adsorption, four different cluster geometries were explored. There are two 2D clusters and two 3D clusters. The relaxed geometries are shown in Figure 7 and Figure 8. The 2D structures are four atoms adsorbed in a line along equivalent sites and in a rhombus shape with metal atoms binding to adjacent, equivalent sites. For the 3D structures, two atoms are adsorbed at equivalent sites, with two atoms atop these to create a 3D rectangle. Similarly, for the tetrahedral geometry, three atoms are adsorbed to the ML, with a fourth atom atop these to create a tetrahedron.

Adsorption of Co at site **atop_Mo** in the 3D tetrahedral configuration (Figure 7G,H,K) is the most favourable geometry with a binding energy of −6.33 eV/atom, with a linear adsorption mode at site **atop_Mo** (Figure 7B) and a 3D tetrahedral configuration at site **hollow** (Figure 7L) showing binding energies of −6.10 and −6.05 eV, respectively. Generally, the 3D rectangle structure is not as favourable, so that the 3D tetrahedral motifs predominate for four-atom clusters, with the 2D linear configurations also favourable.

Of the twelve initial binding configurations, upon relaxation five structures show atoms migrating from their original positions. Three of these are the 3D rectangle configuration, which shows that this geometry is not favourable for Co_4_. At sites **atop_S** and **atop_Mo** (Figure 7G,H), this configuration rearranges to the most favourable tetrahedral geometry with the base atoms adsorbed at site **atop_Mo**. At site **hollow**, one of the atop atoms moves to the surface to adsorb at a site **atop_S**, while the second atop atom migrates to rest between the two base atoms, which remain at site **hollow**, forming a 3D rhombus structure shown in Figure 7I. The remaining rearrangements are discussed in section S1.1.1 of Supporting Information File 1.

Rearrangements of the MoS_2_ ML can also be observed. In the 2D configurations we can clearly see the Co atoms integrating into the S layer of the ML (Figure 7C,F,L). Co atoms from the line configuration at site **hollow** incorporate into the S layer with little movement away from the original lattice, which could be one of the origins for the relative stability of this structure.

For Ru_4_ adsorption, we find that similar to Co_4_, a 3D tetrahedral configuration at site **atop_Mo** is the most favourable geometry with an adsorption energy of −5.14 eV (Figure 8K). Similarly to Co_4_ and Ru_3_, five of the twelve Ru_4_ configurations exhibit atom migration to different sites or cause the ML to rearrange, or in some cases both.

However, in contrast to Co_4_, the tetrahedral configuration is not favoured in these rearrangements. Instead, we observe more structural distortions to the Ru_4_ geometries and the MoS_2_ lattice. In the case of the 3D rectangle at site **atop_S** these distortions cause a transition from a 3D structure to a 2D structure, as shown in Figure 8G. As for adsorptions at site **hollow** discussed previously, the most dramatic distortions to the MoS_2_ ML occur at this site. The line and tetrahedral configurations (Figure 8C,L), in particular, caused strong lattice distortions. While the Ru atoms have remained at their original adsorption sites, several S atoms migrated out of the surface to accommodate their incorporation into the S layer and have formed bonds with the Ru adatoms, creating a Ru_4_S_3_ cluster. More details on the various geometries are given in section S1.1.2 of Supporting Information File 1.

#### Discussion

Comparing the contributions of metal–metal and metal–substrate interaction energies to the total binding energy is an important way of determining a preference towards 2D or 3D growth. With this particular system it is also important to consider that the metal–substrate interaction and the total binding energy reflect any energy changes associated with rearrangements in the MoS_2_ ML. With this in mind there are three different factors that can influence the metal–substrate interaction. These are (a) a lack of metal–metal bonds, (b) ML rearrangements including incorporation of metal atoms into the S layer and (c) formation of metal–S clusters. Incorporation of adatoms into the surface layer is known to increase the metal–substrate interaction, as is the lack of metal–metal bonds [48]. A gain in the metal–substrate interaction energy from formation of a metal–S cluster is due to Equation 2 not accounting for this, meaning that energy contributions from the formation of metal–S clusters are included in the metal–substrate interaction energy. Thus, it cannot be used in a straightforward manner as an indicator of 2D-vs-3D growth for these geometries.

We find that there are five Co*_n_* geometries where the metal–substrate interaction is more favourable than the metal–metal interaction. These are the separated Co_2_ atoms at site **atop_Mo** along with all of the linear site **hollow** adsorptions for Co_3_ and Co_4_ clusters. For these structures the increase in the metal–substrate interaction is caused by the lack of metal–metal bonds. For the site **hollow** structures, the increase in the metal–substrate interaction is due to the incorporation of Co into the S layer as well as the limited metal–metal interactions in a linear structure.

There are ten Ru*_n_* structures where the metal–substrate interaction is more favourable than the metal–metal interaction. However, we observe significant ML distortions for Ru*_n_* adsorption. Hence, this should not be used as the only indicator for potential 2D-vs-3D growth. In these cases, both the metal–substrate and the metal–metal interactions were positive.

Similar to Co*_n_*, the majority of the structures that have a more favourable metal–substrate interaction are those that do not involve any metal–metal bonds or those where the Ru adatoms were incorporated into the S layer. Several of these structures involved the formation of metal–S structures, which can account in part for the larger number of structures with a more favourable metal–substrate interaction.

Bond length analysis shows that the length of Co–Co and Ru–Ru bonds is directly influenced by the geometry of the adsorbed structure. This means that the majority of metal–metal bonds are shorter than the bulk metal–metal distances of 2.48 Å [52] for Co and 2.67 Å [55] for Ru, with some configurations showing longer distances depending on the arrangement of Co and Ru atoms. Co–S and Ru–S bonds change in length depending on the adsorption site, with the shortest bonds measured for site **atop_S** adsorptions. Bonds can also be longer due to structural distortions. Mo–S bonds are generally unaffected or slightly shorter than their equivalent on the bare ML. However, the local distortions described above can cause Mo–S distances to change by between −0.17 and +0.28 Å for Co adsorption and by between −0.26 and +0.23 Å for Ru adsorption. Section S1.5 of Supporting Information File 1 presents a more detailed analysis of the bonding.

Calculating addition energies allows us to study the energy gain as more atoms are added to a structure. Due to the many rearrangements of configurations observed, the energy gain computed from Equation 4 also contains the energy gained from the local atomic rearrangements previously described.

We observe particularly large addition energies for structures such as the Co_4_ line and 3D rectangle configurations, when rearrangements occur to realise a particularly favourable structure. Smaller addition energies are observed for rearrangements to a structure that is less favourable. Where no rearrangement occurs, the addition energies are of similar magnitude for different configurations, indicating that the variation arises only from the structure. Further details on addition energy are given in section S1.2 of Supporting Information File 1.

We now discuss the electronic properties of Co and Ru clusters adsorbed on MoS_2_. From the computed Bader charges, a metallic Co atom has 9.0 valence electrons, while a Co atom is considered oxidised when it has a Bader charge of less than 9.0 electrons. Similarly, metallic Ru has 8.0 valence electrons, and oxidised Ru will have a Bader charge of less than 8.0 electrons.

Analysis of the Bader charges for Co adsorption shows that, in general, atoms bound to the MoS_2_ ML are oxidised. Adatoms that are only bound to other Co atoms remain metallic. Overall oxidation of Co atoms varies with atom coordination.

For Ru we find that atoms are partially oxidised when adsorbing to the MoS_2_ ML, with a computed Bader charge in the range of 7.6 to 7.8 electrons, compared to metallic Ru with 8.0 electrons. As the number of metal adatoms increases, atoms tend to be less oxidised and show more metallic character. Any Ru atom in a 3D configuration that is only adsorbed to other Ru atoms remains metallic with Bader charges of 7.9 to 8.0 electrons. The tetrahedral configuration at site **hollow**, which has formed a Ru_4_S_3_ cluster has a Bader charge of 7.6 electrons for the atop Ru atom that forms new Ru–S bonds. Further details of the Bader charge analysis are given in section S1.3 of Supporting Information File 1.

The changes in charge density are localised around the adatoms and the Mo and S atoms in the immediate neighbourhood of the adatoms. Atoms that were found to be near metallic during the Bader analysis are also found to have somewhat less charge density compared to atoms that were oxidised. There is no distinct difference in how Co and Ru affect the charge density with adsorption to MoS_2_. Examples of the charge density differences are shown in Figure S1 and Figure S2 of Supporting Information File 1, with some additional discussion in section S1.4.

Analysis of DOS plots shows that the Mo d-orbital or the S p-orbital contributions are largely unaffected by adatom adsorption. The metal d-orbital contribution increases for both Co and Ru as more adatoms are added, causing the total DOS to become increasingly more metallic compared to bare MoS_2_, which is a semiconductor. Metal d-orbital states appear in the bandgap for as little as a single adatom. These increase in magnitude as the overall metal contribution increases with added adatoms. Some mid-gap states for Mo d-orbitals and S p-orbitals also begin to appear as more adatoms are added, contributing to the increasingly metallic nature of the system. DOS plots for all configurations are shown in section S3 of Supporting Information File 1.

A brief analysis of the magnetism of Co structures is given in section S5 of Supporting Information File 1.

### Adsorption of Co and Ru clusters on defective MoS_2_

It is well known from both theoretical and experimental studies, that the MoS_2_ ML easily forms S vacancies. To get a first insight into how the presence of such a vacancy might change the interaction between the metal and the ML, we repeat the simulations of the adsorption of single metal atoms and M_4_ structures on a defective MoS_2_ ML with a single S vacancy. This was carried out before in our previous work with Cu adsorption. Using the formation of H_2_S from H_2_, we computed a vacancy formation energy of −6.16 eV, which confirms that defects are easily formed. Furthermore, our results showed that the presence of an S vacancy (giving a concentration of 2% vacancies per supercell) improved Cu adhesion and promoted the formation of 3D clusters [28].

The binding energies, the metal–substrate interaction energies, and the metal–metal interaction energies for all structures on defective MoS_2_ are shown in Table 4, Table 5, and Table 6, respectively. Discussion on bond lengths can be found in section S2.1 of Supporting Information File 1.

**Table 4:** Computed binding energies for Co_1_, Co_4_, Ru_1_ and Ru_4_ on defective MoS_2_ ML for various atom configurations using Equation 1.

No. of metal atoms	Configuration	*E*_bind_/Co-atom [eV]			*E*_bind_/Ru-atom [eV]		
		S_atop	Mo_atop	hollow	S_atop	Mo_atop	hollow

1	—	−6.83	−5.22	−5.12	−5.59	−3.32	−2.91
							
4	line	−5.43	−6.22	—	−4.48	−4.91	−5.16
	rhombus	—	−6.25	−6.64	—	−4.83	−4.33
	3D rectangle	—	−5.78	−6.29	—	−4.89	−4.71
	tetrahedral	—	−6.22	−6.00	—	−4.95	−4.72

**Table 5:** Computed metal–substrate interaction energies Co_4_ and Ru_4_ on defective MoS_2_ ML for various atom configurations using Equation 2.

No. of metal atoms	Configuration	*E*_metal−substrate_/Co-atom [eV]			*E*_metal−substrate_/Ru-atom [eV]		
		S_atop	Mo_atop	hollow	S_atop	Mo_atop	hollow

4	line	−1.79	−2.30	—	−0.16	−2.68	−4.52
	rhombus	—	−1.96	−4.15	—	−2.76	−2.88
	3D rectangle	—	−0.73	−1.58	—	−1.01	−0.70
	tetrahedral	—	−1.37	−1.17	—	−1.60	−1.06

**Table 6:** Computed metal–metal interaction energies for Co_4_ and Ru_4_ on defective MoS_2_ using Equation 3.

No. of metal atoms	Configuration	*E*_interact_/Co-atom [eV]			*E*_interact_/Ru-atom [eV]		
		S_atop	Mo_atop	hollow	S_atop	Mo_atop	hollow

4	line	−4.05	−3.92	—	−2.35	−2.22	−0.64
	rhombus	—	−4.29	−2.49	—	−2.07	−1.45
	3D rectangle	—	−5.05	−4.71	—	−3.88	−4.00
	tetrahedral	—	−4.86	−4.83	—	−3.35	−3.67

**Figure 9:**
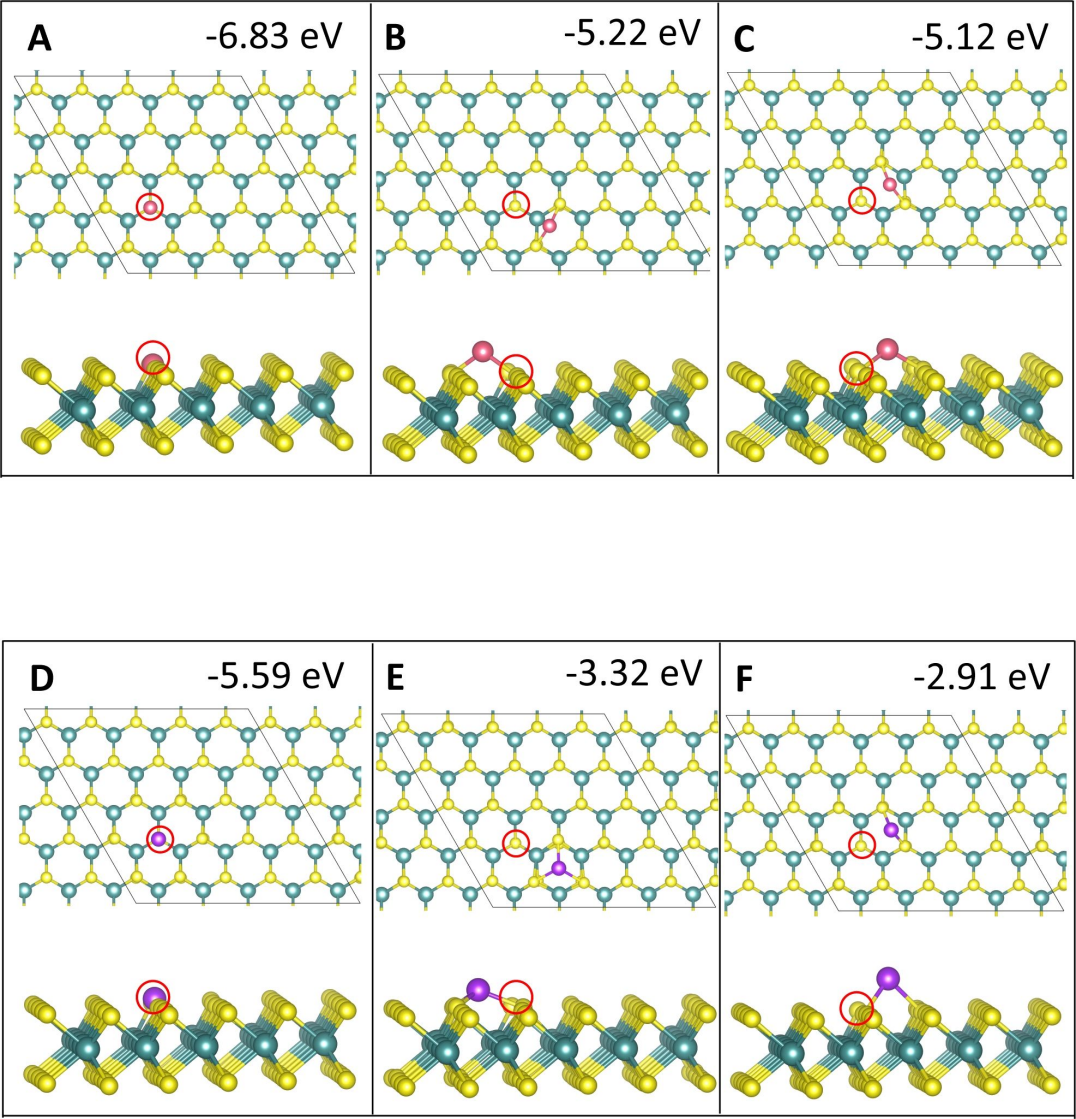
Adsorption modes of single Co (pink) and Ru (purple) atoms on defective MoS_2_. The red circle indicates the location of the S vacancy.

For single-atom adsorption, we find that the metal–ML interaction is stronger at site **atop_S**, where, similarly to Cu [28], Co and Ru fill the vacancy site. This leads to a stronger binding energy by approx. 2 eV for Co and by approx. 3 eV for Ru, compared to the same initial adsorption site on the pristine ML. While, for Cu we found that the metal–ML interaction was enhanced at all sites, for Co and Ru we find that the initial adsorption at sites **atop_Mo** and **hollow** is not stable. For Co, a single atom at either site **atop_Mo** or **hollow** migrates away from its original adsorption site and the vacancy to bridge between two S atoms between site **atop_Mo** and **hollow**. The same occurs for a Ru atom at site **hollow**, while adsorption at site **atop_Mo** leads to the atom migrating to the nearest site **hollow**. These adsorption configurations are less favourable by approximately 0.5 eV, than adsorption at the corresponding sites **atop_Mo** and **hollow** on the pristine ML, and more favourable than adsorptions at site **atop_S** on the pristine ML. The corresponding geometries are shown in Figure 9.

**Figure 10:**
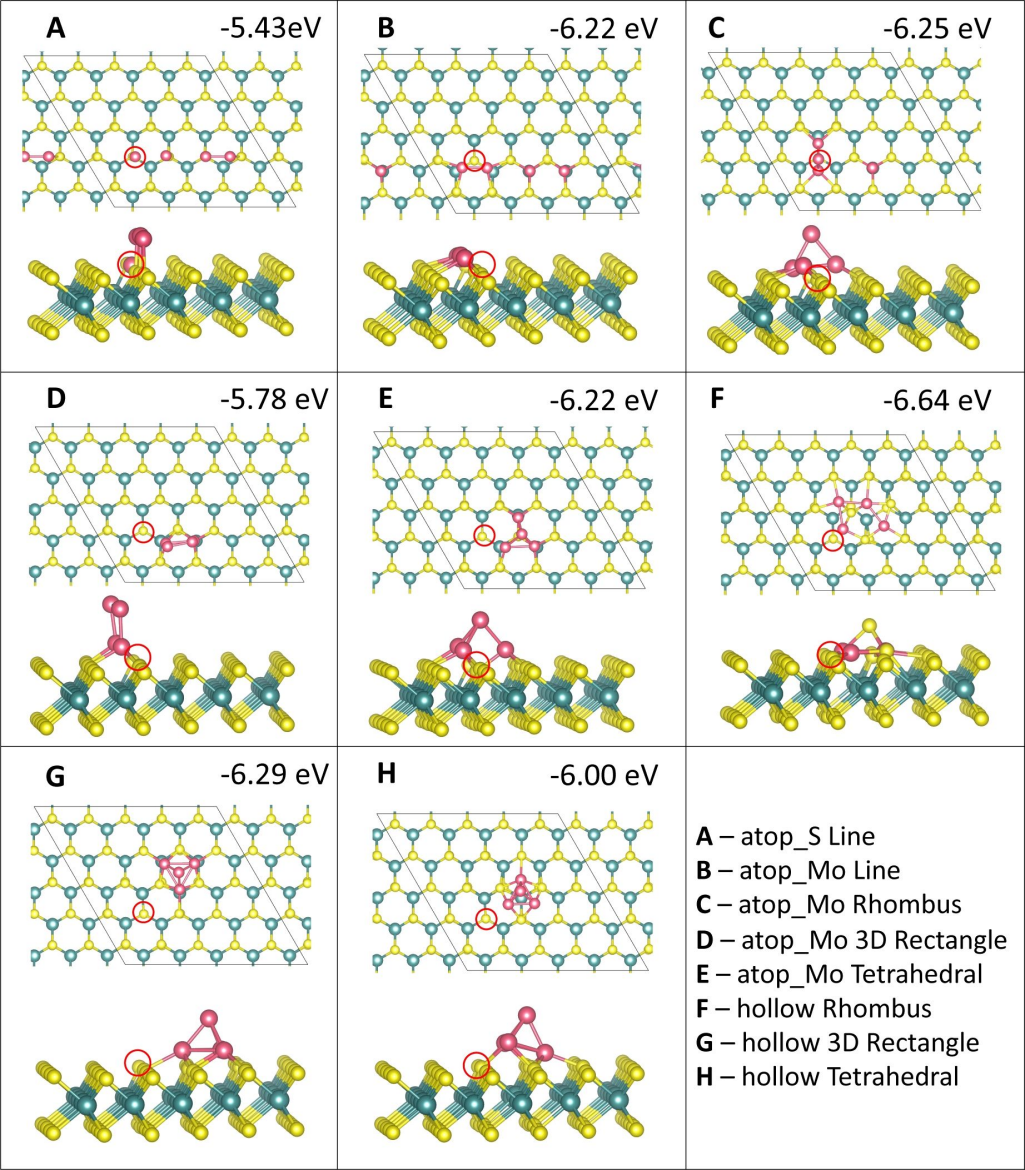
Atomic structure and energies of Co_4_ adsorption modes on defective MoS_2_. The red circle indicates the location of the S vacancy.

For Co_4_, a decrease in the interaction strength is observed for the line configuration at site **atop_S**, and the 3D rectangle at site **atop_Mo**. For the line configuration at site **atop_S**, this is due to the atoms remaining in a line, compared to the migration to a different site that was observed on the pristine surface. Despite the formation of a bond between two of the Co atoms and one of the Co atoms migrating into the vacancy, the binding energy of −5.43 eV is less favourable compared to −6.11 eV on the pristine surface. All geometries for Co_4_ are shown in Figure 10.

An increase in the interaction strength is observed for the line and rhombus configurations at site **atop_Mo** and the rhombus and 3D rectangle configurations at site **hollow**. Depending on the configuration this change can be attributed to bond formation, structural rearrangement compared to the same adsorption configuration on the pristine ML, or in the case of the rhombus at site **hollow**, surface distortion and C*_x_*S*_y_* formation, which also creates an additional S vacancy. The latter is facilitated by the presence of the vacancy and does not occur on the pristine ML.

The presence of the vacancy changes the charge distribution compared to the pristine surface. Atoms near the vacancy are less oxidised (with computed Bader charges of 8.7 to 8.8 electrons) and atoms further away from the vacancy site are more oxidised (Bader charges of 8.5 electrons). The apex atoms in 3D structures are metallic, even for structures where these were slightly oxidised on the pristine surface.

Even though the effects of the vacancy and the adsorption of Co remain localised, the charge density difference shows that the area over which significant changes in charge density take place is larger than on the pristine surface. Visualisations of the charge density differences on the defective ML are included in Figure S1 and Figure S2 of Supporting Information File 1. Metal–metal interaction energies for Co are found to be generally of the same magnitude as on the pristine surface. There is no clear correlation between the binding energy and the metal–metal interaction energy. There are no Co geometries on the defective surface where the metal–substrate interaction is more favourable than the metal–metal interaction. The interaction energy is also not affected by the incorporation of S into the Co cluster as is observed for the rhombus configuration at site **hollow**. This structure has very favourable adhesion to the ML, but the metal–metal interaction energy is similar to that on the pristine surface.

**Figure 11:**
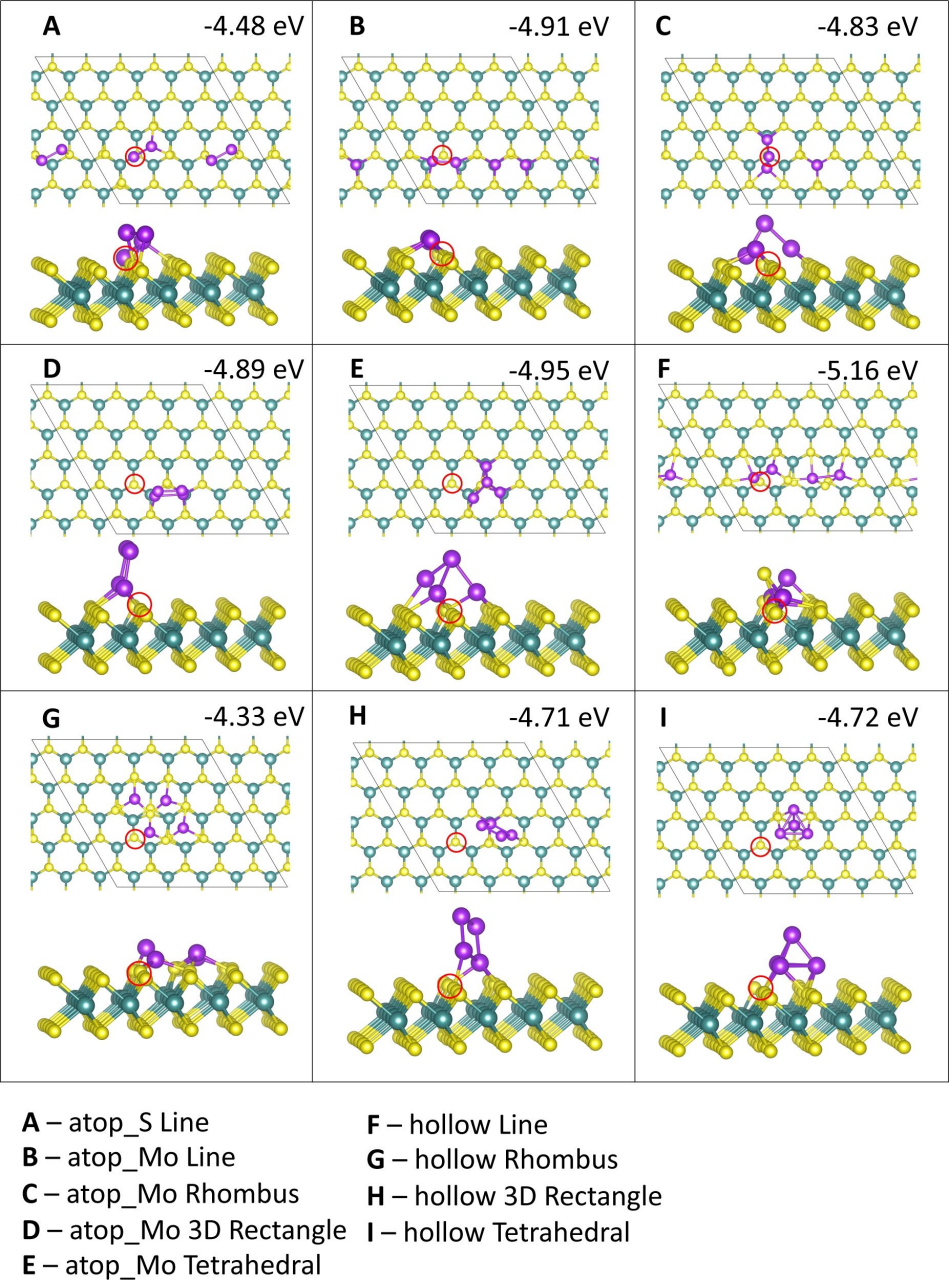
Atomic structure and energies of Ru_4_ adsorption modes on defective MoS_2_. The red circle indicates the location of the S vacancy.

Ru_4_ structures remained largely unchanged compared to the pristine ML. All Ru_4_ geometries are shown in Figure 11. The only decrease in stability is observed for the tetrahedron at site **atop_Mo**, which distorts away from the vacancy. An increase in stability of between 0.1 and 0.4 eV is observed for the line configurations at sites **atop_S** and **atop_Mo**, the rhombus configuration at site **atop_Mo** and the 3D rectangle configuration at site **hollow**.

The line configuration initially at site **atop_S** relaxes to produce two separated Co_2_ clusters. Of the two atoms near the vacancy, one has moved into the vacancy to replace S, while the other is adsorbed at a site **hollow**. The energy gain compared to the pristine surface can be attributed to the additional Ru–Ru bonds as well as the incorporation of Ru into the vacancy, which is always favourable. The line configuration at site **atop_Mo** has the same geometry for Ru_4_ as Co_4_ and Cu_4_[28], with two Ru atoms bridging over the vacancy, as shown in Figure 11B. Similarly, the rhombus configuration at site **atop_Mo** has rearranged to the same 3D triangle over the vacancy with the fourth atom at the nearest site **atop_Mo** that was observed for Co_4_ and Cu_4_ on the defective ML.

Even though the binding energy for the tetrahedron at site **hollow** is similar on the defective ML and the pristine ML, the presence of the vacancy prevents the incorporation of the basal Ru atoms into the S layer. This is the case for all the structures at site **hollow**, which cause less distortion on the defective ML compared to the pristine ML. The exception to this is the line configuration at the **hollow** site (Figure 11F). Here, two Ru_2_ dimers are formed, with a S atom bridging between them, creating a Ru_4_S structure and an additional vacancy in the ML. This is also the most favourable Ru_4_ structure on the defective ML.

Ru atoms are similarly oxidised on both the pristine and the defective ML, although as for Co, atoms near the vacancy tend to be less oxidised with approx. 7.8 electrons. Once again, atop Ru atoms in 3D structures remain metallic, with computed Bader charges of 7.9 to 8.0 electrons. Interestingly, the atop atom in the rearranged rhombus structure at site **atop_Mo** is reduced, with a computed Bader charge of 8.1 electrons, making it the only metal atom in any of the configurations to become reduced. Additional Bader analysis is detailed in section S2.2 of Supporting Information File 1. The charge density difference for Ru on the defective ML is similar to that on the pristine ML (Figure S2G,H of Supporting Information File 1).

The effect of a vacancy on the metal–metal interaction energy of adsorbed Ru structures is significant. For most structures, the magnitude of the metal–metal interaction energies is approximately half that of the pristine ML. This is best illustrated using the example of the rearranged rhombus configuration at site **atop_Mo** as this same rearrangement also occurs for Co. While for Co the interaction energy weakens by approximately 0.7 eV due to the separated fourth atom, for Ru the interaction is weakened by more than 3 eV from −5.29 eV on the pristine surface to −2.07 eV on the defective surface. This change can be attributed to the effect of the vacancy on adsorbed Ru structures. There are some exceptions, where the metal–metal interaction is strengthened compared to the pristine ML. However, this is due to a difference in geometry compared to the pristine surface.

There are three geometries adsorbed on the defective ML that have a metal–substrate interaction more favourable than the metal–metal interaction. These are the linear configurations at site **atop_Mo** and **hollow** and the rhombus at site **hollow**. All three of these structures have none or few metal–metal bonds and both configurations at site **hollow** involve incorporation into the S layer, which increases the strength of the metal–substrate interaction.

### Comparison of interactions of Cu, Co, and Ru with pristine and defective MoS_2_

The interaction of the metals Cu, Co, and Ru with the pristine and defective MoS_2_ ML clearly depends on the nature of the metal. This is most obvious from the magnitude of the binding energies of a single atom. A single Cu atom binds to pristine MoS_2_ with energies ranging from −0.8 to −1.3 eV. The adsorption energy of a Co atom ranges between −4.2 and −5.8 eV, while the adsorption energy of a Ru atom ranges between −2.5 and −3.9 eV.

For metal atoms and dimers, the stability of Cu and Ru adsorption is governed by the adsorption site, with site **atop_Mo** the most favourable site for both. Ru atoms at site **hollow** cause distortions in the ML. For Co, the adsorption site can also influence the stability, with site **atop_Mo** the most favourable overall. Site **atop_Mo** is favoured, as it follows the MoS_2_ lattice structure. We find that this, as well as the symmetrical addition of atoms at the same site instead of non-equivalent sites, is correlated with the strength of adhesion.

As *n* increases, Cu adsorption no longer depends on the adsorption site. Instead, the stability of structures is governed by the number of Cu–Cu bonds. This means that for *n* = 3–4, all geometries have very similar binding energies. Further, for *n* = 4, site **atop_S** is the most favourable. The distance between neighbouring sites **atop_S** is shorter compared to sites **atop_Mo** and **hollow** and the proximity of atoms adsorbed at site **atop_S** facilitates Cu–Cu bond formation [28].

Co adsorption continues to be controlled by the adsorption site for *n* = 3. Different geometries have very similar energies at the same site, with the most favourable adsorption configurations at site **atop_Mo**. Co_4_ prefers tetrahedral geometries on pristine MoS_2_, with the majority of adsorption geometries rearranging to this type of structure. Some of the 2D structures at site **hollow** result in strong distortions, involving metal migration into the S layer, which increases the strength of their interaction with the ML and makes these structures competitive in energy with the tetrahedral geometries.

Similarly to Co, Ru_3_ adsorption is mainly influenced by the adsorption site, with some influences from the overall geometry. This is particularly noticeable at site **atop_S**, where the triangle configuration has a binding energy that is 1.5 eV weaker compared to the other two geometries. This is also clear evidence that Ru atoms prefer to be associated, as the lower binding energy for the triangle at site **atop_S** arises from the separation of the atoms. In contrast, when *n* = 4, Ru structures are more stable when there are minimal distortions to the geometry or the ML, or when Ru atoms incorporate into the S layer and Ru*_x_*S*_y_* structures are formed.

For Cu, Co, and Ru, adsorption of a single atom on the defective ML is most favourable when the metal atom fills the vacancy. Cu adsorption at sites **atop_Mo** and **hollow** is also enhanced, while these adsorption sites become unfavourable for Co and Ru as the adatoms migrate away from the vacancy during the relaxation. Adsorption at the bridge site for Co and at the bridge site and site **hollow** for Ru is less favourable than adsorption on the pristine ML.

The presence of the S vacancy also enhances the binding of Cu_4_, although only a limited number of geometries were stable, while others were repelled from the ML. The stable configurations showed a preference to be 3D. 2D and 3D Co_4_ structures were competitive in energy, with a preference towards structures that incorporated into the S layer. Further, the vacancy facilitated the transfer of an S atom from the ML onto the Co structure to create a Co_4_S cluster, which was also the most favourable Co_4_ structure on the defective ML. This shows that, in the presence of Co, further vacancies can be formed through the transfer of S atoms onto the metal cluster. In contrast, the presence of the vacancy prevented the incorporation of Ru atoms into the S layer to some extent and was also found to weaken metal–metal interactions. However, a Ru_2_S cluster with two adjacent Ru atoms was formed and is the most favourable structure, similar to Co_4_ adsorption.

It is difficult to predict whether a thin film will grow in a 2D or 3D structure, but a useful descriptor is how the metal–substrate interaction compares to the metal–metal interaction and how the total binding energy compares to the bulk metal cohesive energy. While the metal–substrate interaction exceeds the metal–metal interaction energy for several Co*_n_* and Ru*_n_* structures, only Co has a total binding energy that is more favourable than the cohesive energy. The most favourable adsorptions for both Ru and Cu are 1 eV less favourable than their respective cohesive energies [58]. Despite this, we have shown that Ru incorporation into the S layer on the pristine surface and the presence of a vacancy both enhance the Ru–substrate interaction and weaken the metal–metal interaction. It is therefore possible that the presence of more vacancies in the MoS_2_ ML could promote a 2D growth of Ru. Further work including the calculation of the activation energies for 2D or 3D clusters will give a more detailed insight into the processes that control aggregation on the surface. However, this is out of the scope of the current study.

Based on our findings MoS_2_ would be most suitable as a barrier+liner for a Co interconnect, although based on our results, there are some concerns how the transfer of S atoms from the ML to Co*_n_* would affect the purity of the interconnect. Ru on MoS_2_ might be better suited as a catalyst. However further studies involving larger Ru*_n_* structures are needed to determine if the overall strength of the interaction between Ru and MoS_2_ could be enough to prevent agglomeration. As Ru was less likely to incorporate into the ML on a defective ML, growth of Ru on a defective MoS_2_ ML could be suitable for Ru interconnect systems.

## Conclusion

We have presented an extensive study of the interaction of Co*_n_* and Ru*_n_* species, with *n* = 1–4, at a perfect and a defective MoS_2_ monolayer. We have also compared these metals to Cu*_n_* from our earlier work. MoS_2_ is of great interest for the barrier layer in semiconductor devices and as a support in catalysis, while Ru and Co are potential replacements for Cu as the interconnect metal and are used in metal catalysis. Thus, understanding how the metals interact with MoS_2_ is important. We find that the stability of single-atom adsorption follows the trend Co *>* Ru *>* Cu. Furthermore, this trend holds for the adsorption of all metal species, regardless of the number of adatoms. Co typically adsorbs more strongly than Ru to the MoS_2_ ML by up to 2.0 eV, while adsorption is stronger by up to 5.0 eV compared to Cu.

For two- and three-atom nanoclusters on perfect MoS_2_, we find that the preferred adsorption configuration is determined by the adsorption site at the monolayer. Ru*_n_* adsorption is accompanied by notable surface distortions in the monolayer, in particular the migration of sulfur atoms off their original sites as Ru atoms incorporate into the S layer of MoS_2_. Such rearrangements are not seen for Cu or Co. The binding of Cu and Co nanoclusters appears to be driven by the formation of metal–metal and metal-surface bonds, whereas for Ru, the adsorption configuration is the dominant factor.

The four-atom nanoclusters are the first clusters where 2D and 3D configurations can be compared. Co_4_ structures prefer to adsorb in tetrahedral 3D geometries, as is evident from the rearrangement of the atomic structure when Co atoms are close enough that Co–Co bonds can form. However, linear configurations without Co–Co bonds are competitive in energy with these tetrahedral geometries. This likely originates from the incorporation of Co atoms into the S layer in such structures. In turn, this indicates a strong interaction with the MoS_2_ ML, while tetrahedral geometries have an energy gain originating from Co–Co interactions instead. If we compare with Cu, the Co–S bond enthalpy is much higher than the Cu–S bond enthalpy, at 331 kJ/mol and 274.5 kJ/mol respectively (we did not find data for Ru–S bonds), and the Co cohesive energy is larger at 4.39 eV, compared to 3.49 eV for Cu [58].

Ru_4_ does not have the same preference for tetrahedral motifs as Co_4_, despite the tetrahedral configuration at site **atop_Mo** being the most favourable adsorption mode. Nevertheless, clustered geometries are preferred compared to linear adsorption, probably as a result of the larger Ru cohesive energy of 6.74 eV. Structures with minimal distortion are particularly favoured, as well as those where Ru incorporates into the S layer of MoS_2_ and Ru*_x_*S*_y_* clusters are formed.

On the defective ML, adsorption is most favourable at the vacancy site, where single metal atoms fill the missing S site, causing a significant increase in the interaction energy compared to the pristine ML. Adsorption at other sites is less favourable or unstable compared to the pristine ML. 2D and 3D Co_4_ structures are competitive in energy in the presence of a vacancy, with a preference towards those structures that have metal incorporation into the S layer. The vacancy also facilitates the transfer of S atoms onto the Co cluster and thus the formation of additional vacancies.

The most favourable Ru_4_ structure on the defective ML is the line configuration at site **hollow** where transfer of an S atom onto the Ru structure has occurred. Despite this, we find that Ru atoms are less likely to incorporate into the S layer on a defective surface and that the metal–metal interaction energy is weakened, indicating that in the presence of more vacancies 2D growth of Ru should be promoted.

Our overall findings indicate that for Co 2D and 3D cluster adsorption structures are competitive. However, the binding energy of Co on MoS_2_ is significantly more favourable than the cohesive energy of Co. This strong binding energy as well as the favourable metal–substrate interaction should inhibit migration of atoms to form 3D structures during thin film deposition, resulting in a 2D film suitable for interconnect applications, without the need of an additional liner material to promote wetting. This is the subject of further work and will include study of the activation energies for 2D and 3D structures.

In contrast, Ru has a binding energy that is less favourable than its cohesive energy. This suggests that 3D growth Ru on MoS_2_ will be promoted, making this system more suitable for catalysis applications where 3D structures with large surface-to-volume ratios are desired. However, given the several structures for which the metal–substrate interaction is more favourable than the metal–metal interaction and the overall weakening of the metal–metal interactions caused by an S vacancy, a 2D Ru thin film could be formed in the presence of S vacancies.

## Supporting Information

Supporting Information features additional data on geometries, addition energies, Bader analysis, charge density difference, bondlengths, DOS, van der Waals interactions, and Co magnetism.

File 1 Additional experimental data.
