# Supplementary material for: Prediction of Co and Ru nanocluster morphology on 2D MoS2 from interaction energies
Source: Beilstein J Nanotechnol. 2021 Jul 14;12:704–24. doi: 10.3762/bjnano.12.56 (PMC8290098; doi:10.3762/bjnano.12.56)
Supplement: File 1 — Additional experimental data. [file Beilstein_J_Nanotechnol-12-704-s001.pdf]

BEILSTEIN JOURNAL OF NANOTECHNOLOGY

Supporting Information

for

Prediction of Co and Ru nanocluster morphology on 2D MoS_2 from interaction energies

Cara-Lena Nies and Michael Nolan

Beilstein J. Nanotechnol. **2021,** *12,* 704–724. doi:10.3762/bjnano.12.56

Additional experimental data

License and Terms: This is a supporting information file under the terms of the Creative Commons Attribution License (https://creativecommons.org/licenses/by/4.0). Please note that the reuse, redistribution and reproduction in particular requires that the author(s) and source are credited and that individual graphics may be subject to special legal provisions.
The license is subject to the *Beilstein Journal of Nanotechnology* terms and conditions: (https://www.beilstein-journals.org/bjnano/terms)

S1: Pristine MoS_2

S1.1: Geometries

S1.1.1: Co_4 adsorption on pristine MoS_2

The other two configurations with atom migrations are the linear configuration at site **atop_S** and the 2D rhombus configuration at site **atop_Mo**. For the former, one of the atoms migrates from its location at site 1 to bridge between two S atoms half way between site **atop_Mo** and **hollow**, forming a triangle with two of the atoms (see Figure 7A, main text). For the latter, atoms migrate closer to each other during relaxation, with one atom at site **hollow**, another remaining at site **atop_Mo** and the third and fourth atom binding only to the other Co atoms and, thus, forming a 3D rhombus structure (see Figure 7E, main text). These rearrangements show that if Co atoms are close enough to each other that bonds can be formed, 3D clusters form that resemble a tetrahedron.

S1.1.2: Ru_4 adsorption on pristine MoS_2

Two of the atoms in the line configuration at site **atop_S** migrated to a site **hollow**, while the two atoms that were originally atop in the 3D rectangle configuration at site **atop_S** migrated to the ML and adsorbed at site **atop_Mo** on either side of the two base atoms, as shown in Figure 8A and Figure 8G in the main text. The latter structure is also the least favourable geometry for Ru_4, with an adsorption energy of -3.29 eV. Similarly to the migrations observed for its Co_4 counterpart, the rhombus configuration at site **atop_Mo** is distorted with one atom at site **hollow**, one remaining at site **atop_Mo** and the other two atoms slightly removed from their original position at site **atop_Mo** to allow for Ru–Ru bonding (see Figure 8E, main text). One of the atoms has also lifted away from the surface and is thus only bound to other Ru atoms.

S1.2: Addition energies

Calculating addition energies allows us to study the energy gain as more atoms are added to a structure. Due to the many rearrangements of configurations observed, the energy gain computed from Equation 4 also contains the energy gained from the rearrangement. We observe particularly

large addition energies for structures such as the Co_4 line and 3D rectangle configurations, when rearrangements occur to realise a particularly favourable structure. Smaller addition energies are observed for rearrangements to a structure that is less favourable. Where no rearrangement occurs, the addition energies are of similar magnitude for different configurations, indicating that the variation arises only from the structure. For Ru adsorption, the range of addition energies observed is larger than for Co adsorption, as more rearrangements occur. While most of the major geometry rearrangements occurred for structures at site **hollow**, most of the variation in addition energy happens for adsorptions at site **atop_S**. The least favourable addition of −0.52 eV occurs when moving from the site **atop_S** Ru_3 triangle configuration to the site **atop_S** Ru_4 3D rectangle configuration. This is likely caused by the Ru_4 structure migrating from its original configuration to a 2D formation, causing the overall energy gain to be quite small. Other less favourable addition energies are typically associated with structures that are somewhat distorted or have a less favourable binding energy such as the site **atop_Mo** Ru_4 rhombus configuration. Similarly, very favourable addition energies are associated with structures that form Ru–Ru bonds due to addition of a second atom, such as the transition from neighbouring atoms at site **atop_S** to the Ru_3 line at site **atop_S**, or simply where the addition of an atom yields a much more favourable structure as is the case for adding an atom to neighbouring atoms at site **atop_S** to create the Ru_3 3D triangle at site **atop_S**. The two largest addition energies are observed for the addition of an atom to the Ru_3 triangle configuration at site **atop_S** to create the rhombus and tetrahedral configurations at site **atop_S**. These structures have addition energies of −10.75 and 11.11 eV, respectively. This is because the triangle configuration at site **atop_S** is the least favourable Ru_3 structure.

Table S1: Computed addition Energies for each configuration ($M_{n-1} + M \longrightarrow M_n$) using Eq.4. For the "non-equivalent" configurations for two metal atom adsorption, the column "S_atop site" has atoms at sites S_atop and Mo_atop, "Mo_atop site" has atoms at S_atop and hollow, and "hollow site" has atoms at Mo_atop and hollow.

No. metal atoms	Configuration		E_{add}			E_{add}	
		Site 1	Site 2	Site 3	Site 1	Site 2	Site 3
2	neighbouring	—	−6.29	−5.88	—	−4.97	−3.82
	separated	−4.19	−5.81	−5.90	−2.44	−3.92	−4.10
	non-equivalent	−6.91/−5.30	−6.54/−5.20	−4.29/−6.15	−5.52	−4.95	−2.97
3	line	−4.72	−6.12	−6.17	−7.13	−4.52	−5.74
	triangle	−4.18	−6.28	−6.21	−2.61	−5.06	−5.89
	3D triangle	−4.50	−6.06	−5.23	−7.56	−5.26	−5.58
4	line	−7.60	−6.18	−6.17	−4.89	−4.77	−5.80
	rhombus	−7.15	−5.33	−5.81	−10.75	−3.17	−4.09
	3D rectangle	−8.51	−7.17	−7.05	−0.52	−5.58	−5.40
	tetrahedral	−7.11	−6.88	−6.55	−11.11	−6.62	−5.58

S1.3: Bader Analysis

A metallic Co atom has all 9.0 valence electrons, while an atom is considered oxidised when it has a Bader charge of less than 9.0 electrons. Similary, metallic Ru has 8.0 valence electrons, and oxidised Ru will have a Bader charge of less than 8.0 electrons. Analysis of the Bader charges for Co adsorption shows that in general atoms bound to the MoS_2 ML are oxidised, while adatoms that are only bound to other Co atoms remain metallic. A single exception to this was observed for the tetrahedral configuration at site **hollow**. Here, the atop atom is slightly oxidised with 8.7 electrons. The base atoms for this structure are incorporated into the S layer, which could have caused a change to the charge distributions of this structure.

There is some variance in how strongly Co atoms are oxidised, depending on the adsorption site and particular configurations. This means the oxidation is directly influenced by the coordination of each atom, with some correlation between stronger oxidation (Bader charge ~8.5 electrons) and more favourable adsorption energies. Mo atoms are not electronically affected by the presence of

Co. There are some changes to the oxidation state of S atoms around the adsorption site, but the difference is minimal, varying by ±0.1 electrons, depending on the proximity to Co.

For Ru we find that atoms are slightly oxidised when adsorbing to the MoS_2 ML, with a Bader charge of around 7.6 to 7.8 electrons, compared to metallic Ru with 8.0 electrons. As the quantity of metal adatoms increases, atoms tend to be less oxidised, becoming more metallic in nature. Any Ru atom in a 3D configuration that is only adsorbed to other Ru atoms remains metallic with Bader charges of 7.9 to 8.0 electrons. As for Co, the exception to this is the tetrahedral configuration at site **hollow**. Here, three S atoms have moved out of the ML to form an Ru_4S_3 cluster with the adsorbed Ru_4 structure. With a Bader charge of 7.6 electrons we find that the atop Ru has become oxidised through its bond with S atoms. There are no changes to the Mo charges throughout the surface. S is affected by the presence of Ru more than by the presence of Co. Nearby S atoms gain less electrons (6.4 electrons) than those that have distorted from their original lattice position (6.6 electrons) to accommodate Ru adsorption. S atoms that have rearranged and formed Ru–S clusters are found to also be less reduced, typically with about 6.3 electrons, while there is no change to the charge of S atoms further away from the adsorption site (6.5 electrons). These changes in oxidation state are likely related to the coordination environment of the S atoms.

S1.4: Charge density difference

Changes to the charge density for a selection of adsorption geometries are shown in Figure S1 and Figure S2. The changes in charge density are localised around the adatoms and the Mo and S atoms in the immediate vicinity. Atoms that were found to be near metallic during the Bader analysis are also found to have somewhat less charge density compared to atoms that were oxidised. This is especially visible for the Ru_4 line configuration at site **hollow** (see Figure S2E). Here, the four adatoms all have approximately 7.8 electrons, which is reflected in the minimal charge density difference. It is also of note that for this configuration, there is significant distortion of the surrounding S atoms, which are slightly more oxidised compared to other S atoms in the ML. This can be observed in some small charge density differences around these S atoms, but overall, this particular configuration shows very

little changes in charge density, as compared to the other structures. There is no distinct difference in how Co and Ru affect the charge density with adsorption to MoS_2.

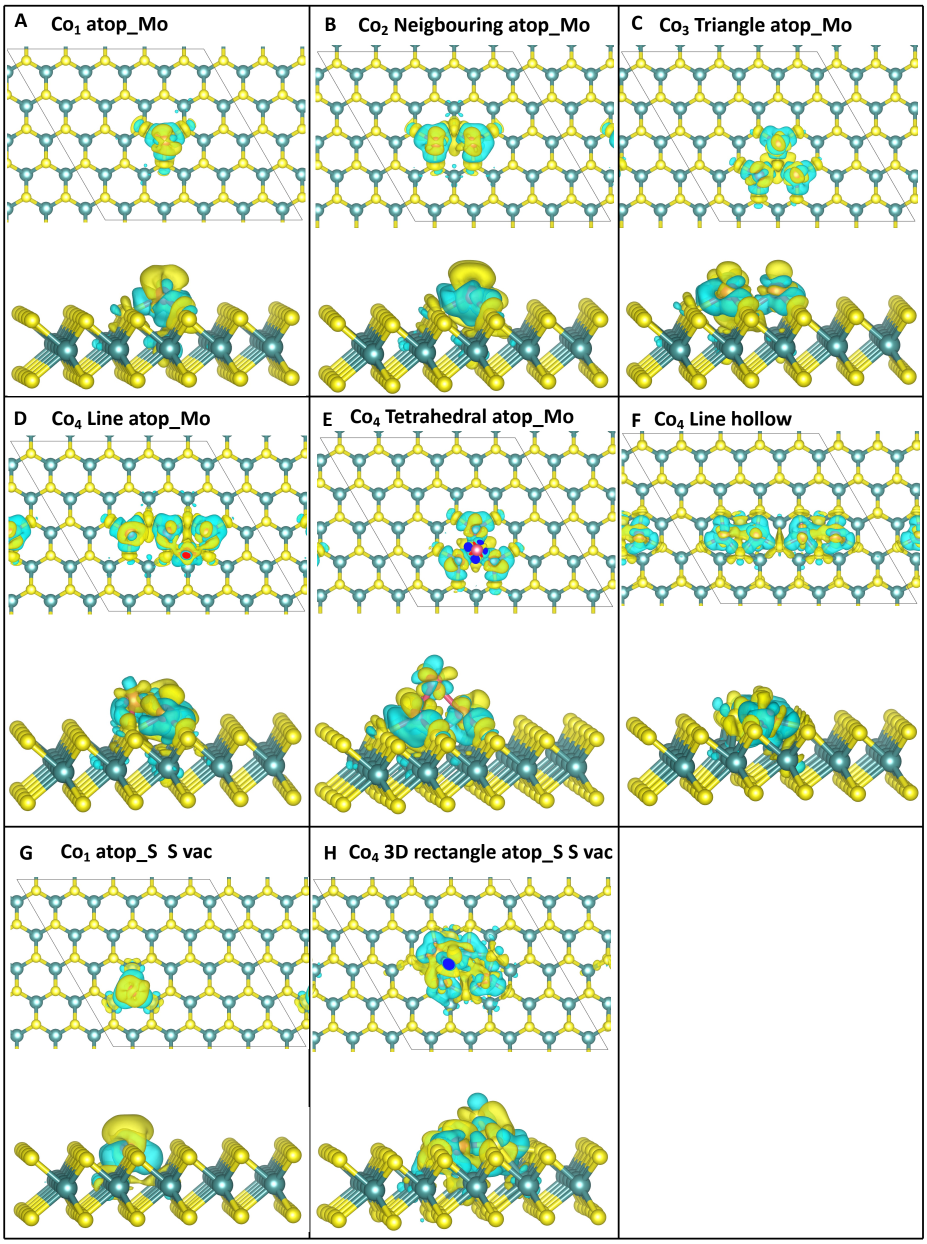

Figure S1: Charge density difference computed for most favourable Co adsorptions.

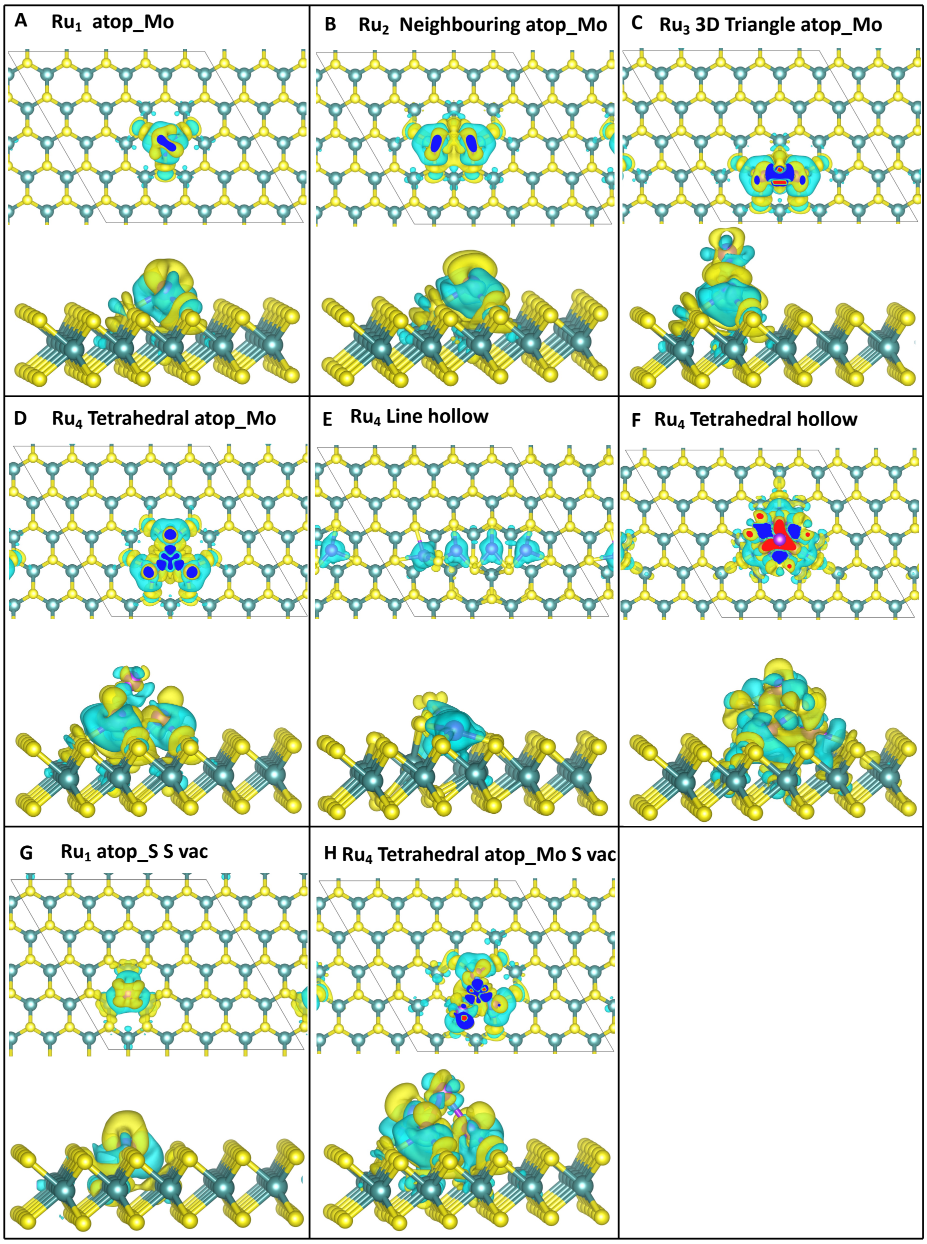

Figure S2: Charge Density Difference computed for most favourable Ru adsorptions.

S1.5: Bondlengths

S1.5.1: One-atom adsorption

We find that for a single adatom, Co–S bonds are shortened to between 1.99 and 2.16 Å compared to 2.31 Å in bulk CoS_2, while Ru–S bonds are shortened to between 2.13 and 2.24 Å compared to 2.37 Å in bulk RuS_2. The shortest metal–S bonds are measured at site **atop_S**. Co–Mo distances are about 0.3 Å longer compared to 2.56 Å in bulk Co_3Mo. There are no Ru–Mo bonds for single-atom adsorption, which is one possible origin for enhanced Co binding, compared to Ru. The length of Mo–S bonds is largely unaffected, with changes around 0.03 Å for sites **atop_S** and **atop_Mo** and of up to 0.1 Å for site **hollow**.

S1.5.2: Two-atom adsorption

Co–Co bonds are between 2.26 and 2.34 Å long, depending on the adsorption sites, which is shorter compared to the Co–Co bulk length of 2.48 Å. As for single-atom adsorption, Co–S atoms are shorter, particularly at site **atop_S**, and range between 1.99 and 2.16 Å. Co–Mo distances continue to be longer compared to the bulk. Mo–S bonds remain largely unaffected, deviating in lengths by −0.01 to +0.2 Å, and both shortest and longest bonds are typically measured for those configurations that have caused slight distortions to the ML. These are neighbouring adsorptions at site **atop_Mo**, adsorptions at site **atop_S** and **hollow** and adsorption at site **atop_Mo** and **hollow**, as shown in Figure 3B, Figure 3H and Figure 3I, in the main text.

Ru–S and Ru–Mo bonds tend to be shorter, compared to their bulk equivalents, and do not appear to be affected by adsorption site. Ru–S bonds range from 2.13 to 2.37 Å, compared to 2.37 Å in the bulk and Ru–Mo bonds range from 2.46 to 2.72 Å, compared to 2.71/2.76 Å in bulk RuMo. Variation within the measured Mo–S bonds are caused by distortions in the lattice structure, due to Ru adsorption. Bonds deviate from the bulk length of 2.42 Å by −0.3 to +0.2 Å.

S1.5.3: Three-atom adsorption

Co–Co bonds were measured to range between 2.08 and 2.64 Å, compared to 2.48 Å in the bulk. The line configuration at site **atop_S** and the 3D triangle at site **atop_Mo** have a mixture of bonds on the shorter and longer end of the range, while the triangle at site **hollow** has only longer Co–Co bonds. For all other configurations, Co–Co bonds are on the shorter end of the range, indicating that Co_3 stability is not influenced by either presence or length of Co–Co bonds.

Co–S bondlengths can be longer or shorter than the bulk length of 2.31 Å, ranging between 2.08 and 2.36 Å depending on the adsorption structure. Most bonds are on the shorter end of the range, particularly for the 2D triangle structures, indicating a potential correlation between shorter Co–S bonds and less favourable structures. Longer bonds occur less frequently, but can be found for all three different adsorption structures.

The majority of Mo–S bonds are within 0.1 Å of the length in the bare ML, which is 2.42 Å. In structures with some surface rearrangements, Mo–S bonds tend to deviate from this near the adsorption sites, and can become somewhat longer compared to elsewhere in the ML, with an increase in length of up to 0.3 Å. These longer Mo–S bonds are also observed for the triangle configuration at site **atop_S** and the 3D triangle for site **atop_Mo**, even though no distortions are visible.

All Ru–Ru bonds measured here are shorter ranging from 2.29 to 2.52 Å, compared to 2.67 Å in the bulk. Ru–S bonds are overall slightly shorter, with some longer bonds measured for configurations with atoms that are quite close to each other, as well as the line configuration at site **hollow**, likely due to the ML distortions observed here. Lengths range between 2.14 and 2.49 Å, compared to 2.37 Å in the bulk. Once again, distortions in the ML are also reflected in some Mo–S bondlengths changes, although the overall deviation of −0.05 to +0.23 Å from the bare ML is quite small.

S1.5.4: Four-atom adsorption

The majority of Co–Co bonds measured are shorter compared to bulk, by up to -0.4 Å. However, half of the Co_4 configurations also have Co–Co bonds that are longer compared to bulk, by up to +0.3 Å.

In particular, this is the case for the various tetrahedral configurations, where due to the geometry some bonds are longer than others.

Co–S bonds are found to be either shorter or similar in length to the bulk length of 2.31 Å, ranging from 2.06 to 2.31 Å. with a single Co–S bond that is 2.54 Å long. This is measured for the 3D rectangle at site **hollow**, and has an increased length due to geometry distortions. Co–Mo distances are consistently longer than the bulk length of 2.55/2.56 Å, with increases of up to 0.35 Å. The majority of Mo–S bonds are the same lengths as the on a bare ML (2.42 Å) or slightly longer in a range up to 2.69 Å, with a small number of bonds that are up to 0.04 Å shorter. The longer bonds are formed due to lattice distortions.

All Ru–Ru bonds measured for Ru_4, are shorter than the bulk length of 2.67 Å, in a range between 2.11 to 2.65 Å. Similarly to what we observe for Co–S bonds, Ru–S bonds are overall shorter than in the bulk length of 2.37 Å, with a few exceptions of longer bonds, in a range between 2.18 and 2.52 Å. These longer bonds are measured for adsorptions at site **hollow** caused by the lattice distortions. Ru–S bonds at site **atop_Mo** are very similar in length to bonds in the bulk, a possible cause for favourable adsorption. Ru–Mo bonds in the bulk are either 2.71 or 2.75 Å long. For Ru_4 adsorption, Ru–Mo bonds are only formed at site **hollow** adsorptions and can be either shorter or longer than the bulk in a range between 2.55 and 2.82 Å. The majority of Mo–S bonds is similar to or slightly longer than the bulk length of 2.42 Å. Longer bonds were measured at site **hollow** and the shortest bonds were measured for site **atop_S**. The overall range was between 2.24 and 2.56 Å.

S2: Defective MoS_2

S2.1: Bondlengths

S2.1.1: Single-atom adsorption

Both Co–S and Ru–S bonds are found to be shorter than the bulk of 2.31 Å and 2.37 Å , respectively, for one-adatom adsorption on the defective ML. Co–S bonds range between 2.02 and 2.05 Å and Ru–S bonds range between 2.18 and 2.28 Å. Co–Mo distances are found to be slightly longer by up

to 0.3 Å than the bulk of 2.55/2.56 Å for adsorption at site **atop_Mo** and **hollow**, while distances at site **atop_S** are shorter than the bulk by about 0.1 /AA. Ru–Mo bonds are only formed at site **atop_S** and range between 2.62 and 2.66 Å, which is slightly shorter than the bulk of 2.71/2.76 Å. As for other sites Mo–S bonds range within 0.1 Å compared to the bare surface of 2.42 Å with slightly longer bonds for Ru adsorption at sites **atop_Mo** and **hollow** and slightly shorter bonds at site **atop_S**.

S2.1.2: Four-atom adsorption

Co–Co bonds are found to be overall similar to bulk of 2.48 Å, while the line configuration at sites **atop_S** and **atop_Mo** and the rhombus configuration at site **atop_Mo** all have slightly shorter Co–Co bonds ranging between 2.14 and 2.38 Å. Co–S bonds are shorter for adsorption at site **atop_S** and **atop_Mo**, ranging between 2.02 and 2.26 Å. For adsorptions at site **hollow**, we find both shorter bonds and slightly longer bonds than the bulk equivalent, ranging between 2.10 and 2.83 Å. Co–Mo distances vary widely depending on the geometry in question, as due to migration into the vacancy and metal incorporation into the S layer, some Co atoms are closer to Mo than others. Lengths range between 2.40 and 2.88 Å , with most on the longer end of the spectrum. Mo–S bonds remain within 0.1 Åof the bare ML, except for the typical elongations and contractions caused by ML distortions.

Ru–Ru bonds are found to be shorter than in Ru bulk of 2.67 Å, ranging between 2.14 and 2.60 Å. Ru–S bonds are overall very similar in lengths to the bulk ranging between 2.10 and 2.53 Å, which makes them slightly longer compared to the pristine surface where the majority of Ru–S bonds was found to be significantly shorter than in the bulk. Ru–Mo distances are mostly similar to bulk, however for configurations with Ru atoms in or near the vacancy site these distances tend to be shorter. The various bondlengths measured range between 2.60 and 2.83 Å. Mo–S bonds are unaffected and typically measured within 0.1 Å of the bare surface reference, as there are no significant lattice distortions for Ru adsorption on the defective ML.

S2.2: Bader Analysis

We find that the presence of the vacancy has a different effect on Co compared to Ru in terms of bader charges. A Co atom adsorbed at site **atop_S** and thus in the vacancy is less oxidised than on the pristine surface. In contrast, Ru adsorbed in the same manner is found to be more oxidised. Co adsorbed at site **atop_Mo** is more oxidised compared to adsorption at site **atop_Mo** on the pristine surface, while Co at site **atop_Mo** and Ru at sites **atop_Mo** and **hollow** have an oxidation state comparable to the same adsorptions on the pristine surface.

Atoms away from the vacancy tend to be more oxidised than those close to it. This is particularly noticeable for the rearranged rhombus configuration at site **atop_Mo**, where the fourth atom that is adsorbed away from the vacancy is more oxidised with 8.5 electrons, and the atoms near the vacancy are less oxidised with 8.8 and 8.7 electrons. A similar effect is observed for most configurations.

As for Co, the separated fourth atom that is adsorbed away from the vacancy is more oxidised than the other Ru atoms. S atoms that were displaced due to adsorption or have moved out of the ML and into the Ru cluster are slightly less reduced, including the S atom incorporated with Ru for the line configuration at site **hollow**, which has 6.4 electrons. The oxidation state of Mo is not affected by Ru adsorption.

The S atom that has moved out of the surface for the rhombus configuration at site **hollow** is less reduced than other S atoms, with 6.4 electrons, and S atoms surrounding the adsorption site are somewhat more reduced than others in the ML with 6.6 electrons. As on the pristine surface, the oxidation state of Mo atoms does not change, either from Co adsorption or the presence of the vacancy.

S3: DOS

Shown below are density of state plots for all metal adsorptions on both pristine and defective MoS_2.

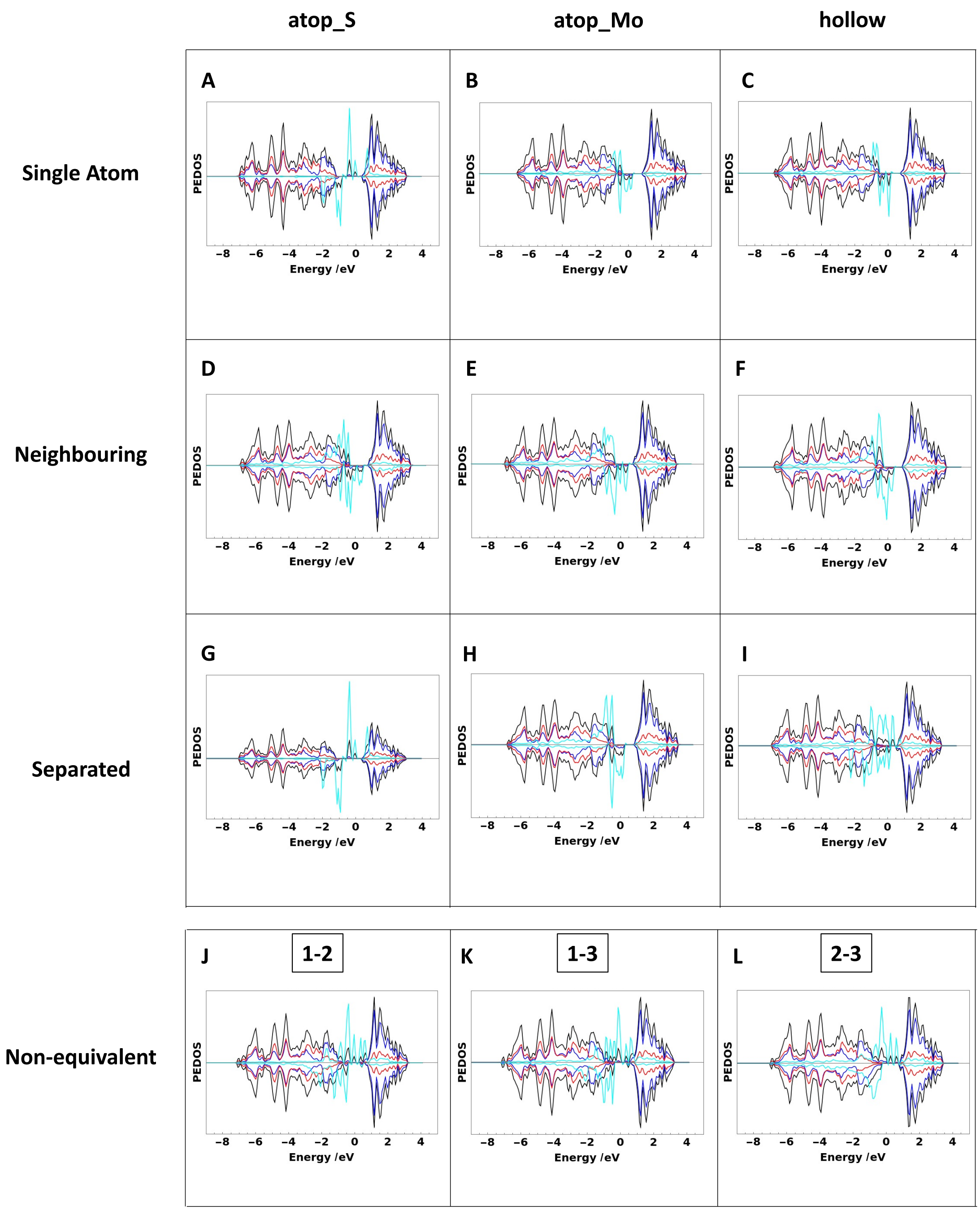

Figure S3: PEDOS for Co and Co_2 adsorption on MoS_2. Black - total DOS; blue - Mo d-orbital contribution; red - S p-orbital contribution; cyan - Co d-orbital contribution

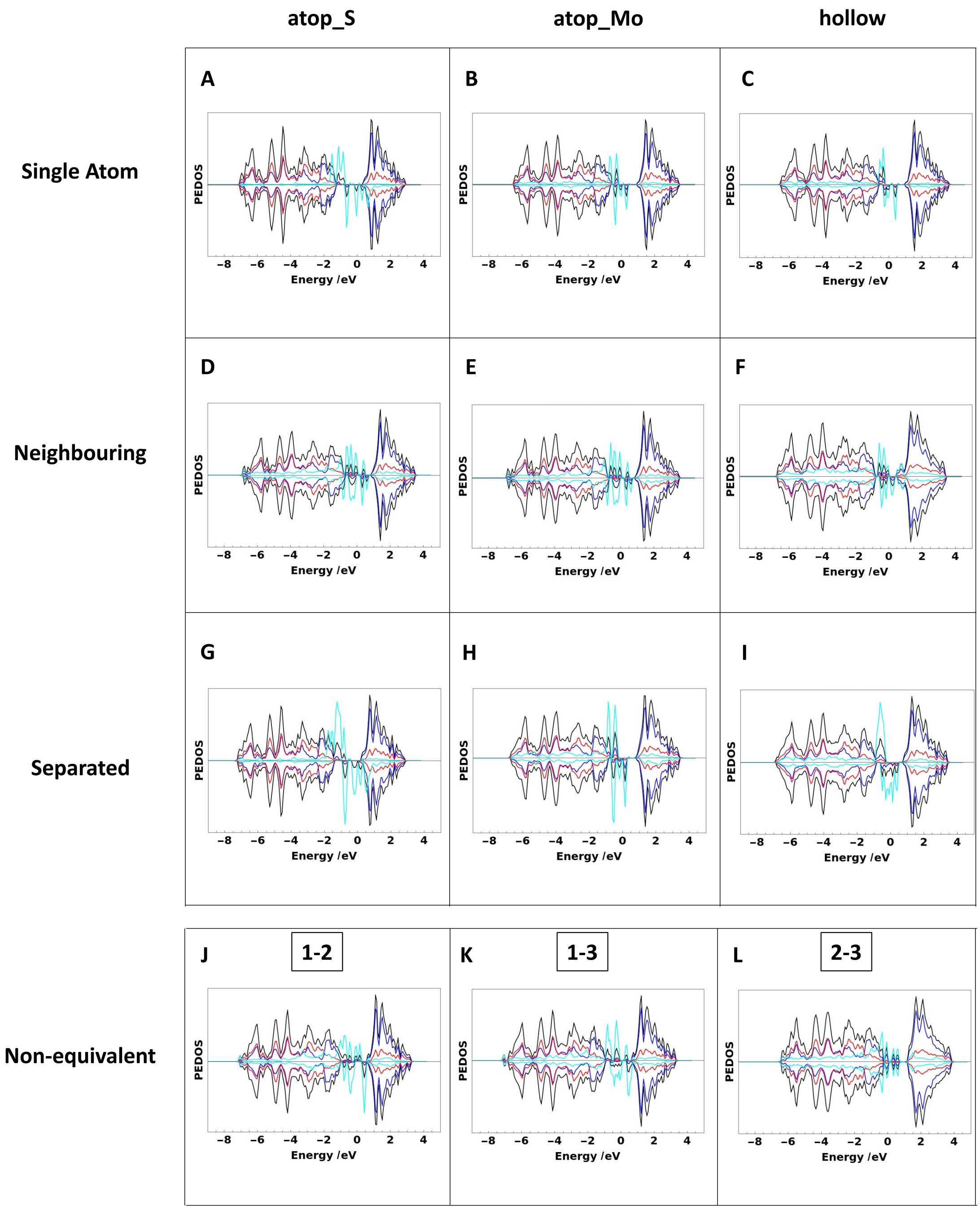

Figure S4: PEDOS for Ru and Ru_2 adsorption on MoS_2. Black - total DOS; blue - Mo d-orbital contribution; red - S p-orbital contribution; cyan - Ru d-orbital contribution

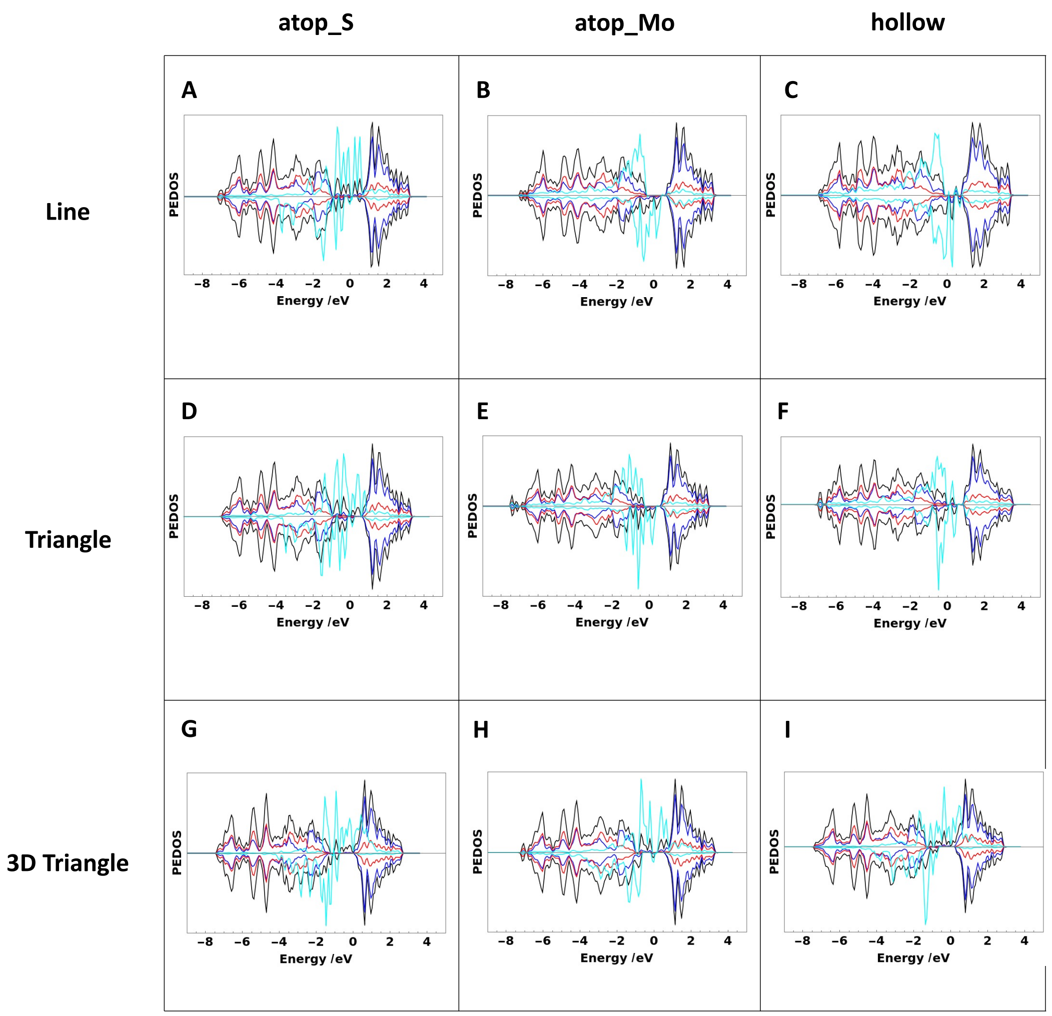

Figure S5: PEDOS for Co_3 adsorption on MoS_2. Black - total DOS; blue - Mo d-orbital contribution; red - S p-orbital contribution; cyan - Co d-orbital contribution

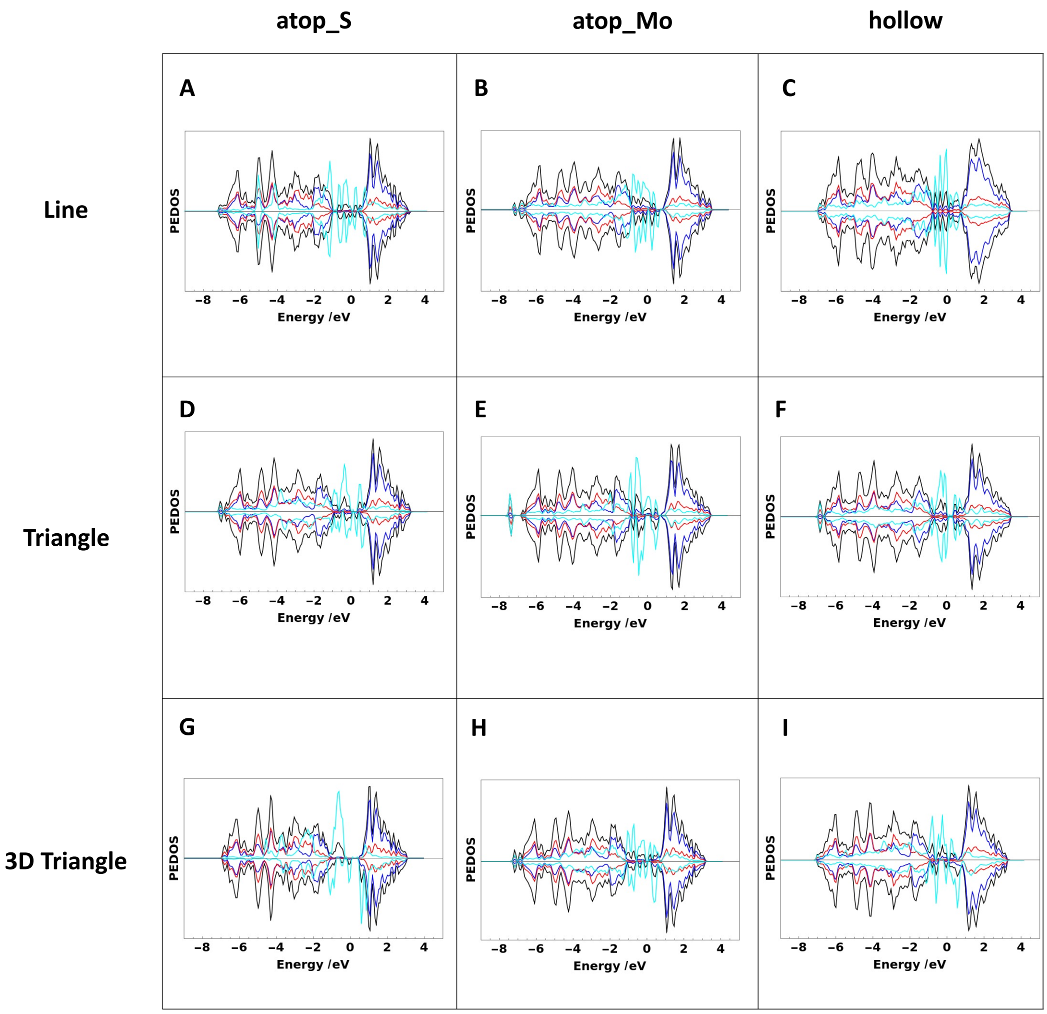

Figure S6: PEDOS for Ru_3 adsorption on MoS_2. Black - total DOS; blue - Mo d-orbital contribution; red - S p-orbital contribution; cyan - Ru d-orbital contribution

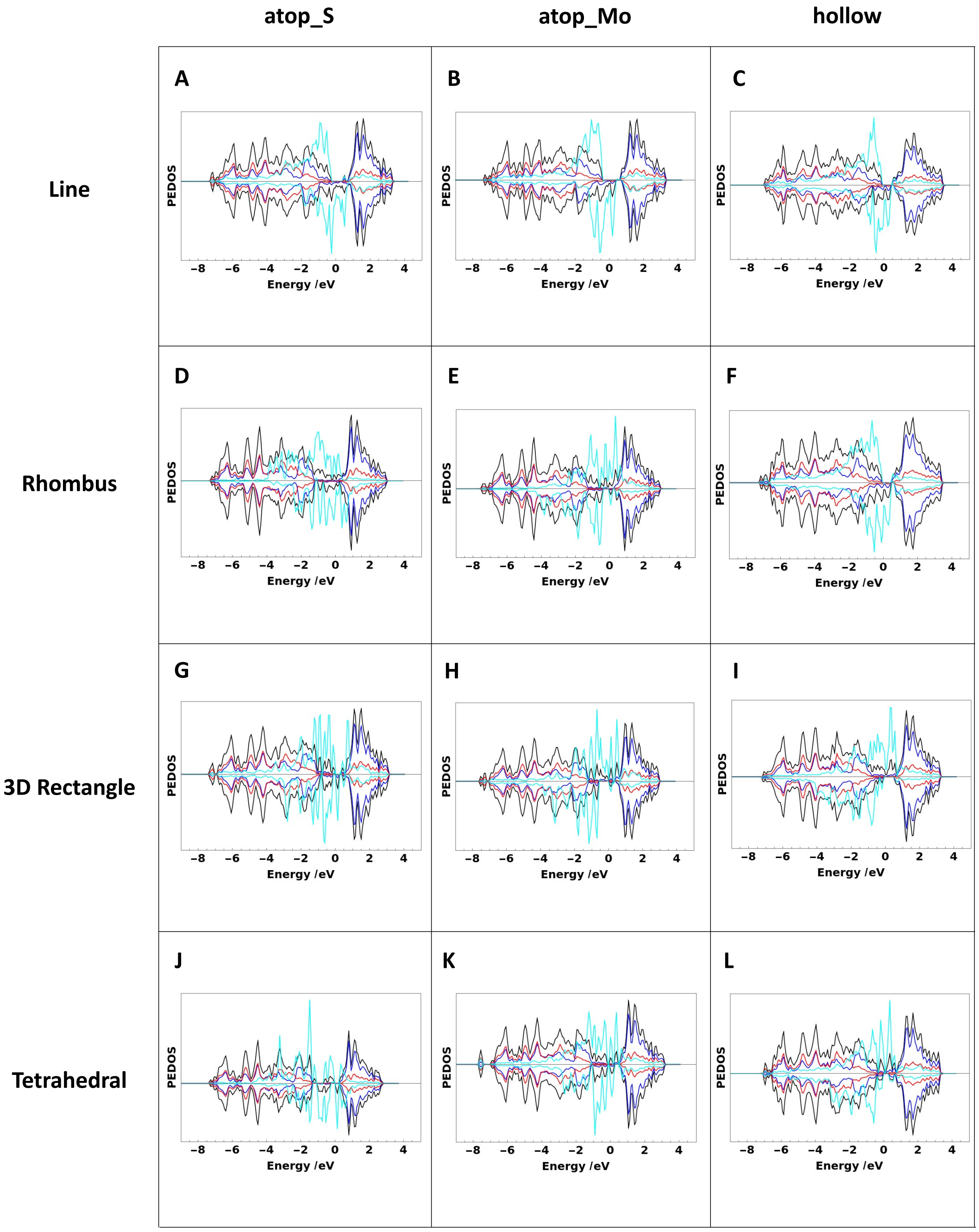

Figure S7: PEDOS for Co_4 adsorption on MoS_2. Black - total DOS; blue - Mo d-orbital contribution; red - S p-orbital contribution; cyan - Co d-orbital contribution

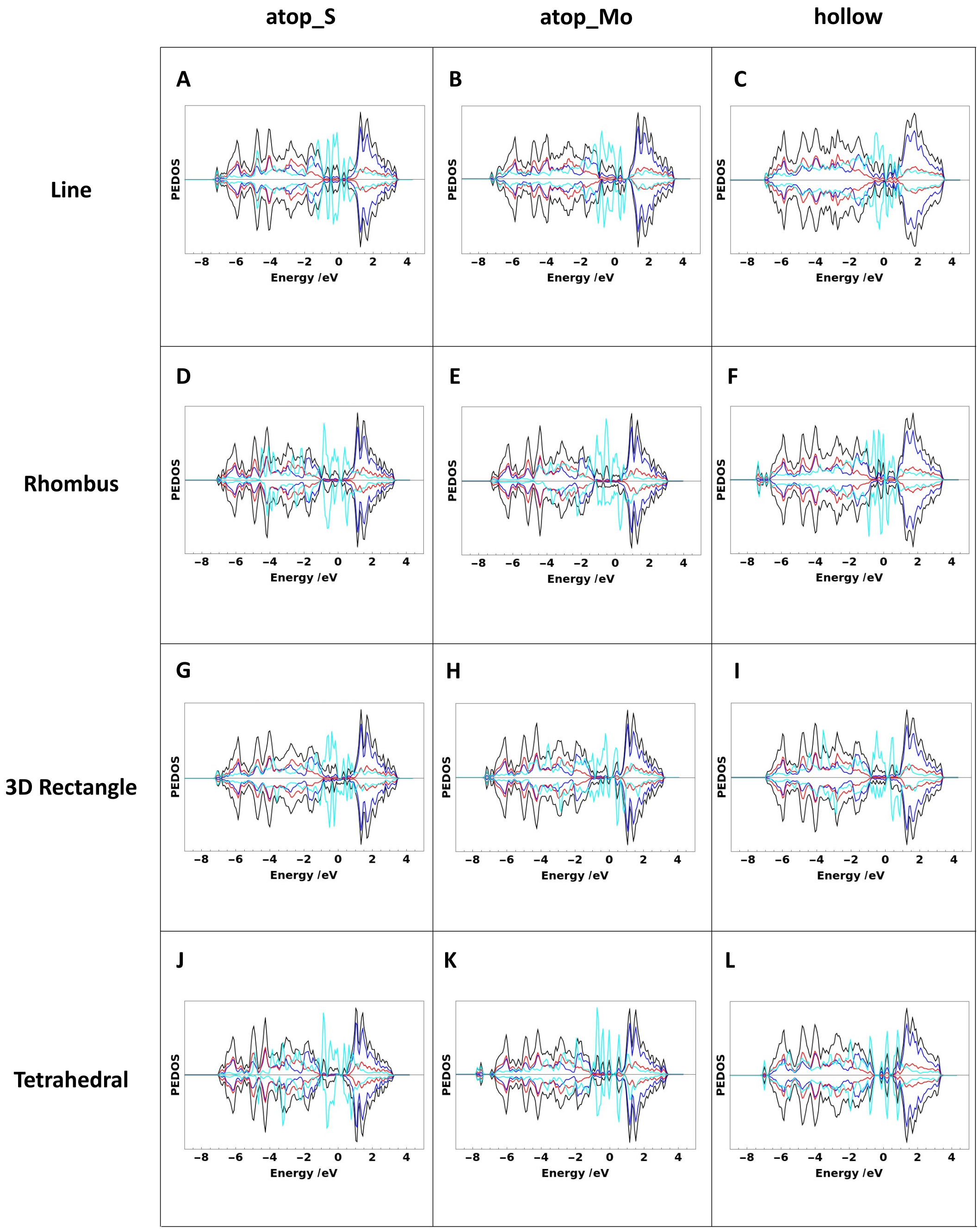

Figure S8: PEDOS for Ru_4 adsorption on MoS_2. Black - total DOS; blue - Mo d-orbital contribution; red - S p-orbital contribution; cyan - Ru d-orbital contribution

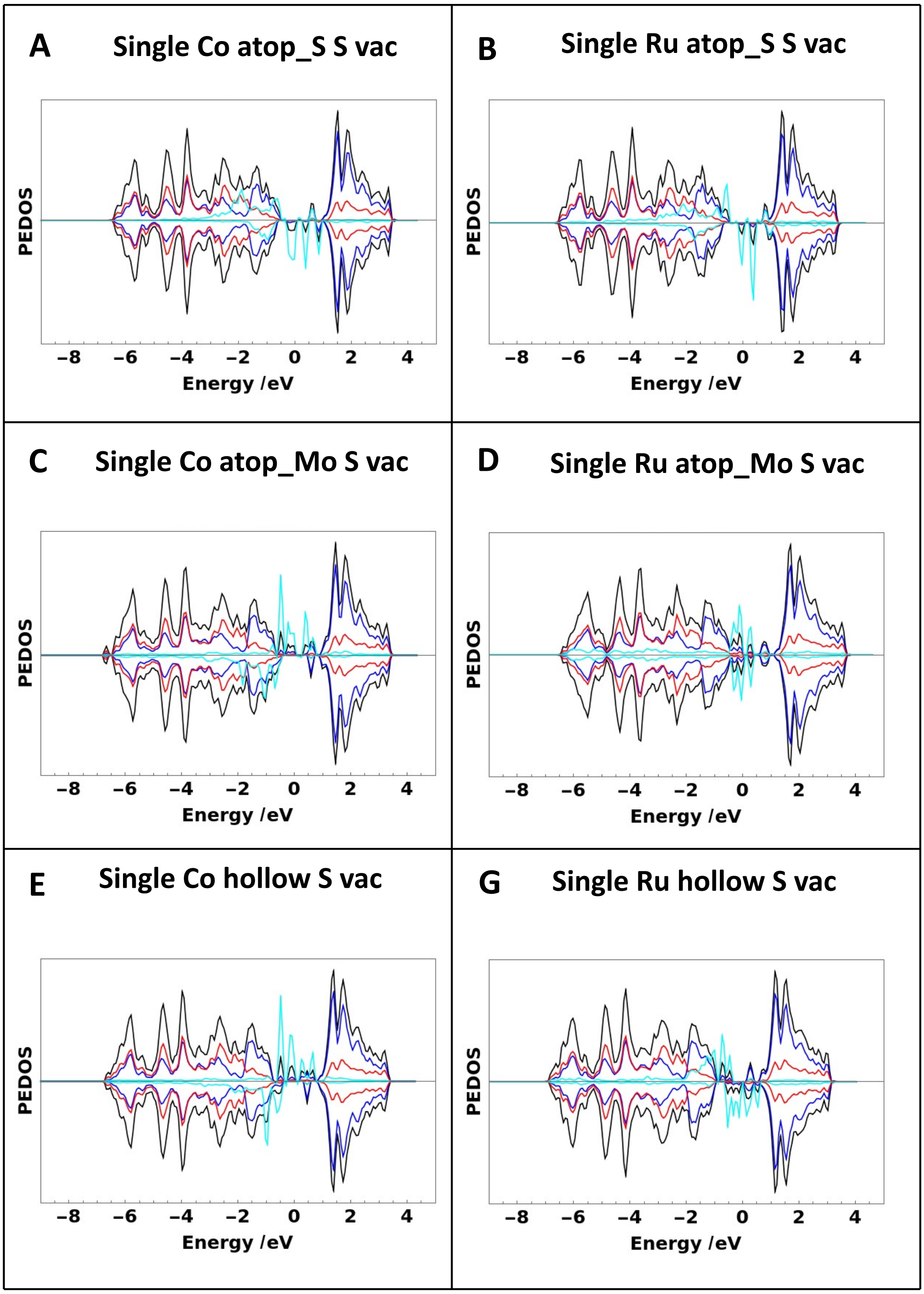

Figure S9: PEDOS for single Co and Ru atom adsorption on defective MoS_2. Black - total DOS; blue - Mo d-orbital contribution; red - S p-orbital contribution; cyan - metal d-orbital contribution

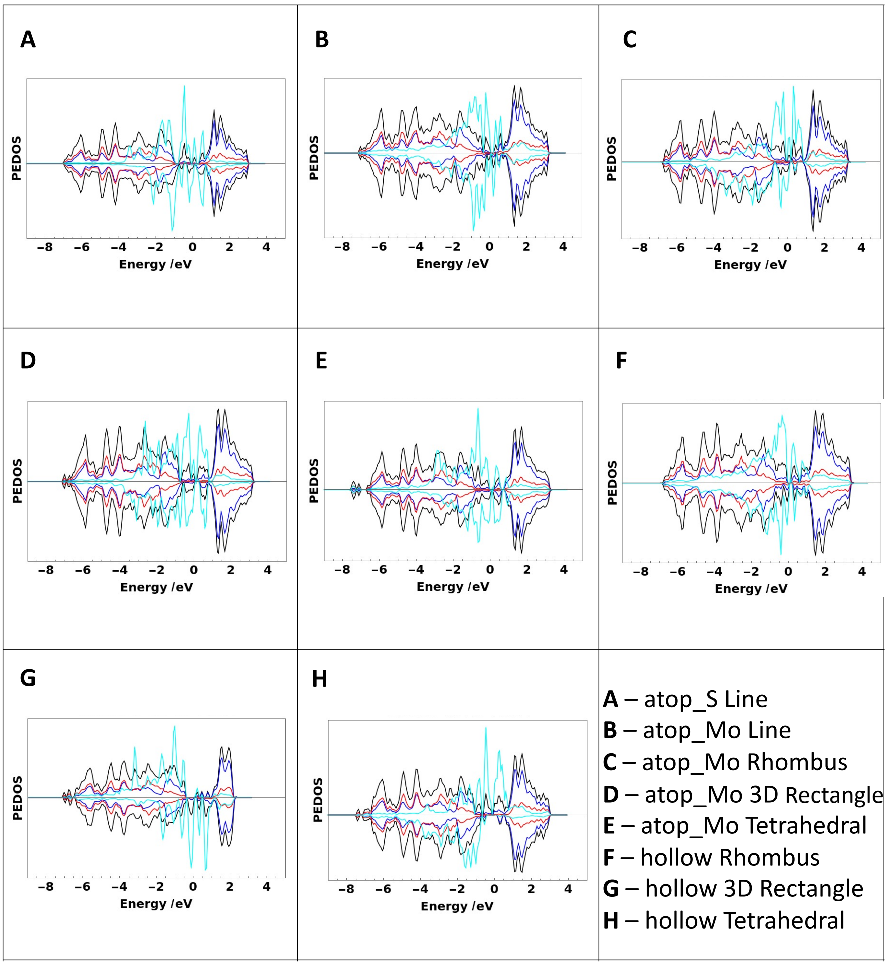

Figure S10: PEDOS for Co_4 adsorption on defective MoS_2. Black - total DOS; blue - Mo d-orbital contribution; red - S p-orbital contribution; cyan - Co d-orbital contribution

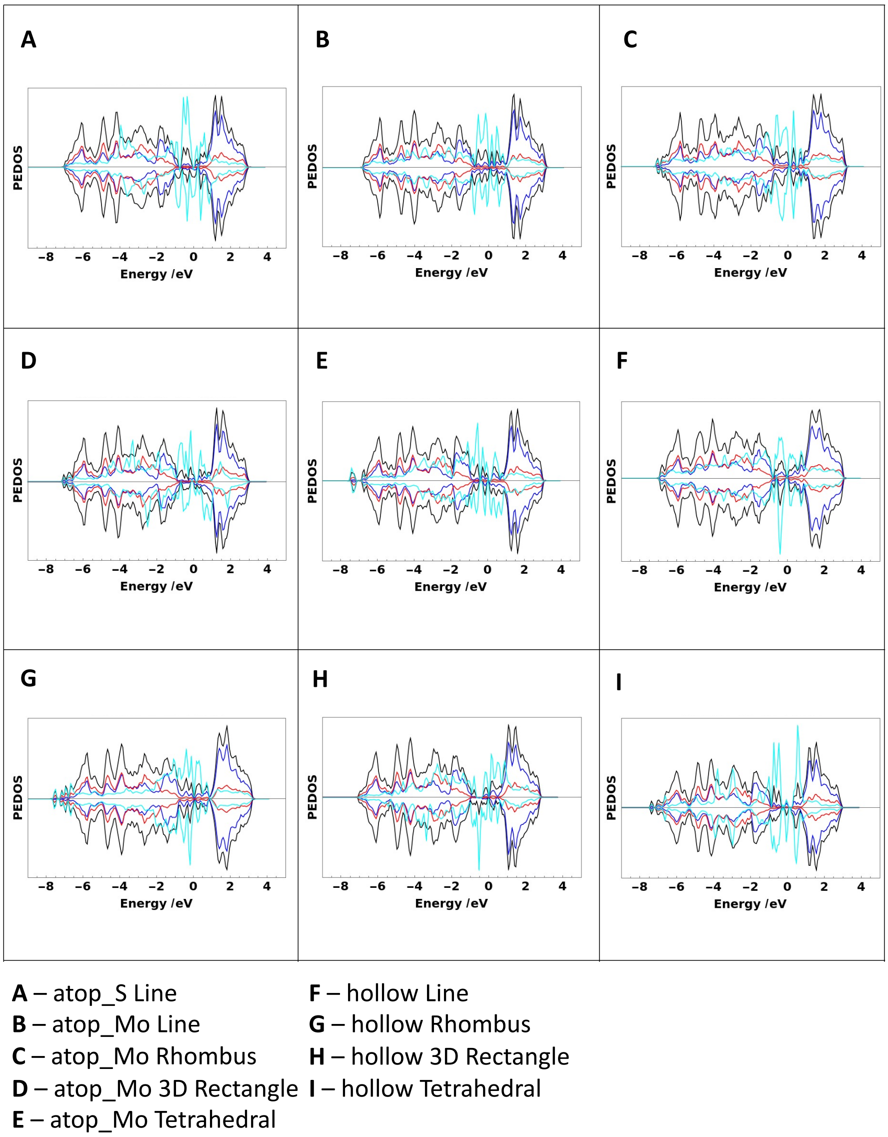

Figure S11: PEDOS for Ru_4 adsorption on defective MoS_2. Black - total DOS; blue - Mo d-orbital contribution; red - S p-orbital contribution; cyan - Ru d-orbital contribution

S4: Impact of van der Waals Interactions

We compared the impact of applying vdW corrections for five Co and five Ru geometries. Nine of the ten structures tested showed no changes to geometry and a consistent increase of the binding energy by ca. 3 eV for Co and ca. 4 eV for Ru, as shown in Table S2 and Table S3. The Co **atop_Mo** rhombus structure rearranges into a tetrahedral structure. Overall, there is no considerable impact on the geometries or energies of the Co and Ru clusters on MoS_2, indicating that they do not play a significant role in the system and can therefore be omitted.

Table S2: Binding energies without and with vdW corrections for selected Co structures.

Geometry	E_{bind}/Co[eV]	E_{bind}/Co[eV] with vdW	Changes with vdW
Line atop_Mo	−6.10	−9.71	None
Tetrahedron atop_Mo	−6.32	−9.83	None
Rhombus atop_Mo	−5.93	−9.86	Relaxed to tetrahedron
Line atop_Mo S vac	−6.22	−9.78	None
Tetrahedron atop_Mo S vac	−6.22	−9.68	None

Table S3: Binding energies without and with vdW corrections for selected Ru structures.

Geometry	E_{bind}/Ru [eV]	E_{bind}/Ru [eV] with vdW	Changes with vdW
3D rectangle atop_Mo	−4.93	−8.43	None
Tetrahedron atop_Mo	−5.14	−8.68	None
Line hollow	−4.76	−8.31	None
Line atop_Mo S vac	−4.91	−8.54	None
Tetrahedron atop_Mo S vac	−4.95	−8.40	None

Magnetism of Co structures

We find that for single atoms, those Co atoms adsorbed at **atop_Mo** and **hollow** sites have a magnetisation of $1\,\mu_B$, which is expected as Co has an uneven number of valence electrons. However, for a single atom **atop_S**, the magnetisation increases to $1.5\mu_B$. This is most likely due to the single Co–S bond at this site, compared to the three Co–S bonds per Co atom at the other two sites. For two Co atoms this effect is balanced by the addition of a second **atop_S** atom, leaving both atoms with a magnetisation of $1\,\mu_B$. Adsorption of atoms at separated sites or non-equivalent sites causes an unequal distribution of magnetic moments, although the total magnetisation remains at

approximately 2 μ_B. For Co_3 and Co_4 clusters the magnetisation becomes quite complicated with large fluctuations in the total magnetism as well as the magnetism of individual atoms. There is no clear correlation between structure stability and magnetic moment. We also observe different distributions of magnetic moments for structures which relaxed to the same geometry. This could be a cause as to why these structures do not have the exact same binding energies.
